# Chorionic Gonadotropin Beta 7 is a marker of immune evasion in cancer

**DOI:** 10.1101/2025.05.28.656535

**Published:** 2025-06-02

**Authors:** Siegen A. McKellar, Jose Mario Bello Pineda, Rohan Lattupally, Amy S. Codd, Evan W. Newell, Sydney X. Lu, Robert K. Bradley

**Affiliations:** 1 Computational Biology Program, Public Health Sciences Division, Fred Hutchinson Cancer Center, Seattle, WA, USA.; 2 Basic Sciences Division, Fred Hutchinson Cancer Center, Seattle, WA, USA.; 3 Medical Scientist Training Program, University of Washington, Seattle, WA, USA.; 4 Molecular and Cellular Biology Graduate Program, University of Washington, Seattle, WA, USA.; 5 Department of Genome Sciences, University of Washington, Seattle, WA, USA.; 6 Division of Hematology, Stanford Medical School, Palo Alto, CA, USA.; 7 Vaccine and Infectious Disease Division, Fred Hutchinson Cancer Center, Seattle, WA, USA.

**Keywords:** CGB7, chorionic gonadotropin beta, beta-hCG, immune checkpoint inhibition, immune evasion, metastatic cancer

## Abstract

Human chorionic gonadotropin beta (beta-hCG) is an oncofetal antigen expressed by trophoblast cells of the placenta, with minimal expression in adult somatic tissues. Numerous studies have demonstrated that beta-hCG-encoding genes are expressed in various cancers, but expression of these genes (*CGB3*, *CGB5*, *CGB7*, and *CGB8*) across diverse cancers has not been systematically evaluated. Here, we report that CGB genes are more widely expressed across diverse cancer types than previously appreciated and that secreted beta-hCG is readily detected. In particular, CGB genes are expressed in the majority of urothelial bladder cancers, where *CGB7* is most frequently expressed and significantly associated with an immunosuppressed tumor microenvironment, including decreased CD8^+^ T cell infiltration. Multiple CGB genes are associated with failure to respond to immune checkpoint inhibitor (ICI) therapy, and *CGB7* is particularly strongly predictive of poor prognosis. Overall, our findings indicate that beta-hCG is a clinically accessible, predictive biomarker of immunotherapeutic response.

## INTRODUCTION

Oncofetal antigens, or proteins that are canonically expressed in immune-permissive developmental contexts and are re-expressed in cancer, have garnered interest for decades as both tumor-specific biomarkers and attractive targets for anti-cancer therapeutics. Notable examples of clinically utilized oncofetal proteins include carcinoembryonic antigen (CEA) and alpha-fetoprotein (AFP). CEA is elevated in multiple malignancies and is commonly used as a biomarker of colorectal cancer, while AFP is a diagnostic marker of testicular cancer and hepatocellular carcinoma (Lazaro-Gorines et al., 2019; Terentiev et al., 2013). Due to the tumor specificity of these markers, they are both being actively investigated as a target antigen for cell therapies such as AFP-targeted CAR-T cell therapies (Sandberg et al., 2022; Munson et al., 2022).

Human Chorionic Gonadotropin Beta (beta-hCG), the beta subunit of essential heterodimeric developmental glycohormone human Chorionic Gonadotropin (hCG), is an oncofetal antigen expressed primarily in trophoblast cells during embryogenesis and reexpressed in multiple cancer types (Lapthorn et al., 1994; Douglas et al., 2014; Iles 2007). Detection of beta-hCG immunoreactivity by enzyme linked immunosorbent assay (ELISA) is the basis of modern clinical tests for pregnancy and trophoblastic disease. Elevated beta-hCG has also been observed in both trophoblastic and non-trophoblastic cancers including germ cell tumors such as choriocarcinoma, urothelial bladder cancer, lung cancer, head and neck cancer, breast cancer, cervical cancer, ovarian cancer, colorectal cancer, endometrial cancer, renal cancer, prostate cancer, and pancreatic cancer (Douglas et al., 2014; Iles 2007). Highlighting the extensive and cancer-specific expression pattern of genes encoding beta-hCG, Chew et al. developed a cancer-specificity scoring metric to screen for cancer-specific genes expressed across multiple cancer types and identified *CGB5*, one of the genes encoding beta-hCG proteins, as the gene exhibiting the strongest pan-cancer signal (Chew et al., 2019).

Beta-hCG expression and the presence of trophoblast-like cells on histology are hallmarks of aberrant trophoblastic differentiation, which can occur in non-choriocarcinomas such as urothelial bladder cancer. Trophoblastic differentiation is associated with poor prognosis and therapeutic resistance (Chang et al., 2024; Cheng et al., 2021). beta-hCG expression alone in many cancer types is associated with poor prognosis (Iles 2007).

hCG is a hormone composed of an alpha subunit encoded by *CGA* and a beta subunit encoded by one of four highly homologous genes: *CGB3* (also known as *CGB*), *CGB5*, *CGB7*, and *CGB8*. Functioning at the maternal-fetal interface, it is essential in decidualization and the maintenance of early pregnancy via both endocrine and paracrine signaling mechanisms. hCG carries out endocrine effects as a driver of progesterone production and exerts paracrine effects that facilitate invasion (Fluhr et al., 2008; Guibourdenche et al., 2010), angiogenesis (Zygmunt et al., 2002; Berndt et al., 2006; Berndt et al., 2009), and maternal immunotolerance (Schumacher et al., 2009; Schumacher et al., 2013; Poloski et al., 2016; Dauven et al., 2016; Schumacher et al., 2017). hCG belongs to the gonadotropin family of hormones, which includes thyroid stimulating hormone (TSH), follicle stimulating hormone (FSH), and luteinizing hormone (LH). This hormone family shares a common alpha subunit, CGA. hCG, TSH, FSH, and LH have essential, but distinct, functions in pregnancy, metabolism, and in development and fertility respectively, with functional specificity conferred by unique beta subunits that possess varying degrees of homology. It is intriguing that upregulation of these specificity-conferring genes encoding beta-hCG proteins is observed in cancer.

Beta-hCG proteins are encoded by four highly homologous primate-specific coding genes arranged in tandem on chromosome 19: *CGB3, CGB5*, *CGB7*, and *CGB8*. This is in contrast with *CGB1* and *CGB2*, which have historically been classified as pseudogenes due to the presence of frameshifts that disrupt their open reading frames. *CGB3*, *CGB5*, and *CGB8* encode identical proteins classified as type 2 CGB proteins. The CGB7 protein differs by three amino acids and encodes a type 1 CGB protein. *CGB7* expression is also observed in some extra-trophoblast tissues and is uniquely regulated (Bellet et al., 1997; Sohr et al., 2011; Zimmermann et al., 2012). We refer to this collection of genes as CGB genes.

As detection and level of CGB protein is currently clinically utilized for pregnancy testing and as a clinical-grade biomarker of trophoblastic disease and germ cell tumors, we sought to explore the potential of CGB proteins as cancer biomarkers. While numerous reports suggest that expression of CGB genes may be valuable cancer biomarkers and prognostic factors, the extent to which each CGB gene is expressed in diverse cancers has not yet been systematically evaluated using the large cohorts of cancer patients available today. Furthermore, the quantitative prognostic utility of CGB expression and the specific cancer phenotypes with which CGB expression is associated remain incompletely understood, particularly across diverse cancer types.

Here, we systematically assess expression of CGB genes across diverse cancer types and reveal that all CGB genes are pervasively expressed across many cancer types, including urothelial cancer, with *CGB7* most frequently expressed. We confirm that CGB genes are transcribed and proteins are detectable as secreted proteins from cancer cells by isoform-specific qRT-PCR and bulk CGB ELISA. We determine that *CGB7* expression is strongly associated with hallmarks of an immunosuppressed tumor microenvironment by re-analyzing RNA-sequencing data, CD8+ T cell infiltration data as measured by immunohistochemistry (IHC), and tumor subtyping data from a cohort of advanced urothelial carcinoma patients. Kaplan-Meier survival analyses elucidate an association between *CGB7* expression and decreased survival probability in a cohort of advanced urothelial carcinoma patients treated with PD-L1 inhibition immunotherapy, which is supported by an association between *CGB7* expression and progressive disease in this cohort. Finally, we propose *CGB7* as a prognostic marker with strong predictive value for response to immune checkpoint inhibition via a random survival forest machine learning model of patient survival.

## RESULTS

### CGB genes are expressed in almost all cancer types

**Figure 1:**
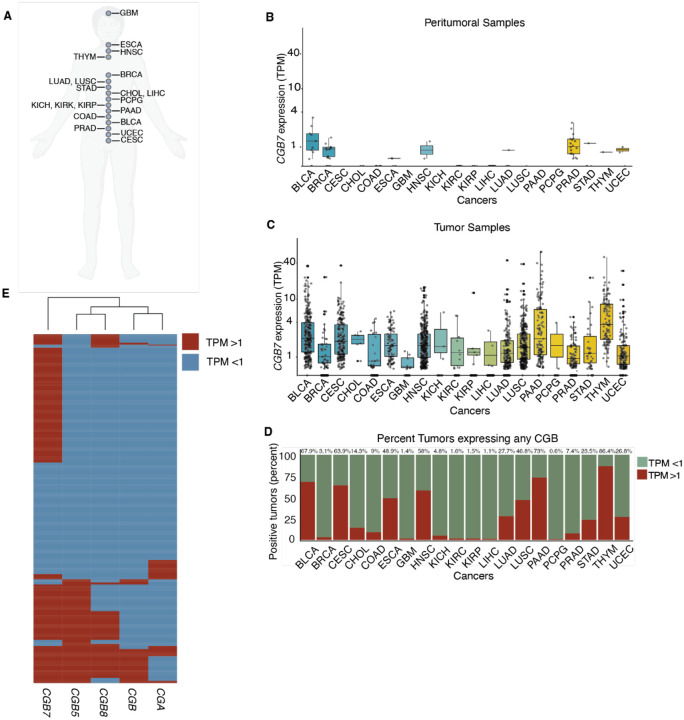
CGB is expressed in multiple cancer types. (**A**) selection of 20 unique cancer mRNA sequencing datasets from The Cancer Genome Atlas (TCGA). *CGB7* mRNA expression in healthy peritumoral tissue samples (**B**), and matched tumor tissue samples (**C**) across 20 cancer types. Cancer type reflects the site of the primary tumor. (**D**) Fraction of tumors across 20 cancer types expressing at least one CGB gene with TPM >1. Percent positive is annotated above each cancer type. (**E**) Expression status of *CGB3*, *CGB5*, *CGB7*, *CGB8*, and *CGA* in tumors from the TCGA bladder cancer (BLCA) dataset. Expressed genes are defined as TPM > 1, non-expressed genes are defined as TPM < 1. TPM = transcripts per million. Data re-analyzed from the TCGA.

We sought to systematically evaluate the expression patterns of each functional CGB gene (*CGB3, CGB5, CGB7*, and *CGB8*) across 20 distinct cancer types utilizing gene expression data from The Cancer Genome Atlas (TCGA) (Figure 1A). We confined this analysis to the 20 tumor types with at least one peritumoral control tissue sample in order to be able to accurately assess cancer specificity of CGB expression. Comparing expression of each CGB gene in tumor and matched normal peritumoral tissues across these datasets elucidated extensive cancer-specific expression of *CGB3, CGB5, CGB7*, and *CGB8* across cancer types (Figure 1B, Figure 1C, Figure S1D–K).

Our results both confirm and notably extend previous reports of CGB expression in cancers as well as identify CGB expression in tumor types in which it has not been previously reported. For example, Cook et al. reports mRNA expression of CGB genes in 30% (n=70) of bladder cancers (Cook 2000). Hotakainen et al. reports mRNA expression of CGB genes in 50% (n=84) of the transitional cell carcinoma cases and in none of the healthy controls assayed (n=15) (Hotakainen 2002). Our new analyses reveal that nearly 68% of such tumors express one or more CGB genes at the mRNA level, while our analyses also reveal common CGB expression in tumor types such as thymoma, which has not been previously reported to our knowledge. Conversely, though *CGA* is expressed across numerous cancers, it is also expressed more frequently in numerous normal tissues, and thus expression of *CGA* does not share a cancer specific expression profile (Figure S1A–B). This is consistent with the pleiotropy of *CGA*, where CGA is a subunit of all gonadotropin hormones, including thyroid stimulating hormone (TSH), follicle stimulating hormone (FSH), and luteinizing hormone (LH), which are expressed outside of development in healthy somatic tissues. Together, these data highlight the specific upregulation of CGB genes in cancer. We conclude that CGB genes are specifically and pervasively expressed in cancers and hypothesize that this specific upregulation reflects a potential novel function of CGB proteins in cancer.

We next identified cancer types that exhibited particularly high frequencies of expression of at least one CGB gene. The three cancer types with highest expression of any CGB are thymoma, pancreatic adenocarcinoma, and urothelial carcinoma (Figure 1D). Trophoblastic differentiation and beta-hCG expression in urothelial cancer have been recognized for decades (Moutzouris 1993, Dirnhofer 1998, Przybycin 2020, Cheng 2021). As urothelial cancer exhibits low frequency of *CGA* expression in our analyses despite the high frequency of expression of CGB genes–highlighting the specificity of CGB gene expression in this cancer type–we selected urothelial cancer as a model to further explore the effects of CGB expression on cancer (Figure 1D, S1A).

Of the four CGB genes, CGB7 is expressed most frequently across cancer types and in urothelial cancer (Figure S2, Figure S1J, Figure S1K, Figure S1L, Figure S1M). We therefore sought to further explore the expression pattern of CGB7 in urothelial carcinoma. We first wondered how frequently *CGB7* is coexpressed with *CGB3*, *CGB5*, *CGB8*, and *CGA* in urothelial carcinoma. Further evaluation of expression of these genes in the TCGA urothelial carcinoma cohort revealed that while tumors tend to co-express *CGB3*, *CGB5*, and *CGB8, CGB7*-expressing tumors cluster independently, suggesting that *CGB7* is less frequently co-expressed with *CGB3, CGB5*, and *CGB8* (Figure 1E). We conclude that *CGB7* exhibits a unique expression pattern in urothelial cancer and is expressed more frequently than *CGB3, CGB5*, and *CGB8* in this dataset (Figure 1E). This is consistent with a previous report of type I CGBs being the predominantly expressed type at the mRNA level in renal cell carcinoma samples (Hotakainen et al., 2006). Furthermore, *CGB7* is frequently expressed independently of *CGA*, suggesting a potential beta-subunit specific effect independent of the hCG heterodimer (Figure 1E). These findings regarding the unique expression pattern of *CGB7* are particularly notable as *CGB7* differs from *CGB3, CGB5*, and *CGB8* in sequence, tissue expression profile, and the sequences of its untranslated regulatory regions (Bellet et al., 1997; Sohr et al., 2011; Zimmermann et al., 2012).

### CGB proteins are secreted by urothelial cancer cells

The comprehensive RNA-seq data provided by the TCGA allows for systematic analysis of CGB gene expression, but these data do not directly address the question of whether expressed CGB mRNA is translated into protein. We therefore directly tested whether CGB protein is produced and secreted by urothelial cancer. We focused on urothelial cancer given the particularly high and frequent expression of CGB that we observed in this tumor type.

We first sought to validate that CGB genes are expressed in human urothelial cancer cells with an orthogonal assay and confirmed that this expression can be reversed by gene silencing. We utilized gene expression data from the Cancer Cell Line Encyclopedia (CCLE) to stratify human urothelial cancer cell lines by CGB expression (Supplemental Table 1). As in human tumor samples, urothelial cancer cell lines exhibit a range of expression of CGB genes (Table 1). Interestingly, *CGB3*, *CGB5*, and *CGB8* frequently exhibited markedly higher expression than did *CGB7* in these cell lines – in contrast to the primary tumor mRNA expression data from the TCGA, where all CGB genes were expressed at a similar level (Table 1). However, this observation is consistent with prior findings that type II CGBs (CGB3, CGB5, and CGB8) exhibited higher expression than type I CGBs (CGB7) in urothelial cancer (Hotakainen et al., 2007). *CGA* expression was low or absent from the majority of these cell lines (Table 1).

**Figure 2:**
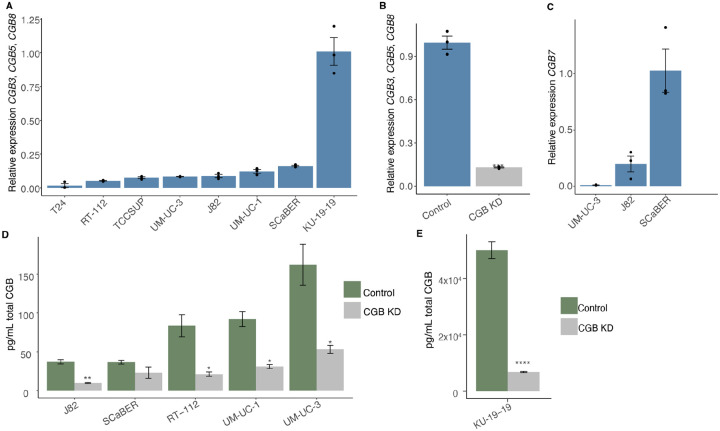
CGB is expressed in multiple human urothelial carcinoma cell lines. (**A**) Expression of bulk *CGB3*, *CGB5*, and *CGB8* mRNA normalized to *GAPDH* quantified by RT-qPCR in human urothelial carcinoma cell lines. Expression is relative to the cell line with the highest expression. Data in Figure 2A–Source Data 2A file. (**B**) Expression of *CGB3*, *CGB5*, and *CGB8* mRNA normalized to *GAPDH* quantified by RT-qPCR in J82 cells treated with a non-targeting siRNA pool or an siRNA pool targeting all CGB genes. Expression is relative to the cell line with the highest expression. P-value calculated via welch two sample t-test. Data in Figure 2B–Source Data 2B file. (**C**) Expression of *CGB7* mRNA normalized to *GAPDH* quantified by RT-qPCR in human urothelial carcinoma cell lines. Expression is relative to the cell line with the highest expression. (**D**) Secretion of total CGB protein quantified by ELISA in human urothelial carcinoma cell lines treated with a non-targeting siRNA pool or an siRNA pool targeting all CGB genes. pg/mL CGB protein shown per 10,000 cells. P-value calculated via welch two sample t-test. Data in Figure 2D–Source Data 2D file. (**E**) Secretion of total CGB protein quantified by ELISA in KU-19–19 cells treated with a non-targeting siRNA pool or an siRNA pool targeting all CGB genes. pg/mL CGB protein shown per 10,000 cells. P-value calculated via welch two sample t-test. All experiments run in biological and technical triplicate; all error bars represent SE; *p < 0.5; **p < 0.01; ****p < 0.0001.

We selected seven urothelial cancer cell lines with high summed CGB expression and one cell line with undetectable CGB expression as examples of CGB-positive and CGB-negative tumors to validate CGB gene expression and protein levels *in vitro*. All cell lines selected are negative for *CGA* expression at the mRNA level. We observed expression of *CGB3, CGB5*, and *CGB8* across the seven positive cell lines surveyed, but not in the negative cell line (Figure 2A). Treatment of J82 cells with a pan-CGB targeting siRNA pool depleted signal from the CGB3/5/8-specific probe in J82 cells (Figure 2B), confirming that the detected mRNA signal arises from expression of CGB genes. We observed clear but low *CGB7* expression in one of the seven assayed cell lines (SCaBER cells; Figure 2C). *CGB7* expression is too low to be reliably detected in the remaining cell lines not shown. This is supported by the lower expression of *CGB7* relative to other CGB genes that we observed in gene expression data from CCLE data (Table 1).

We next validated that CGB proteins are translated and secreted as free beta subunits and that the signal arises from CGB mRNA. Previously, secretion of CGB proteins from several urothelial cancer cell lines was shown (Iles et al., 1987). Iles et al. did not confirm reversibility of signal with knockdown of CGB genes, so we sought to confirm specificity of detection with pan-CGB gene knockdown. We selected J82, SCaBER, RT-112, UM-UC-1, UM-UC-3, and KU-19–19 cells given both their high expression of CGB genes and absent *CGA* expression by mRNA analysis and performed ELISA to detect total CGB protein from supernatant (Figure 2D, 2E). These data confirmed that CGB protein is readily produced and detectable as a secreted protein from all cell lines, with KU-19–19 cells producing particularly notable amounts. As *CGA* is not appreciably expressed in these cell lines (Table 1), we infer that CGB proteins are stable and secreted unbound to CGA. Specificity of the assay was further supported by targeted knock down of bulk CGB genes, resulting in a significant decrease in detected CGB protein (Figure 2D, 2E).

We conclude that both type-I-encoding (*CGB7*) and type-II-encoding (*CGB3, CGB5*, and *CGB8*) CGB genes are expressed in human urothelial cancer cells and that CGB proteins are stable as free beta subunits and are secreted from cancer cells.

### *CGB7* expression is associated with reduced anti-tumor immune activity

**Figure 3:**
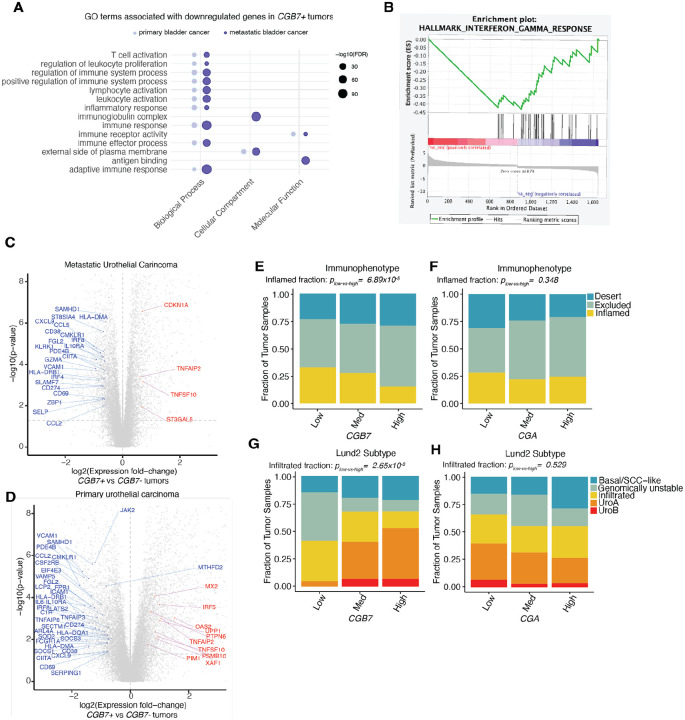
CGB expression correlates with altered immune infiltrate. (**A**) Immune-associated Gene Ontology terms associated with genes downregulated in *CGB7+* tumors in metastatic urothelial cancer (data re-analyzed from Mariathasan et al., 2018) and in primary urothelial cancer (data re-analyzed from TCGA BLCA cohort). *CGB7*+: >1 TPM. *CGB7*−: <0.25 TPM. (**B**) Interferon Gamma response genes are negatively correlated with *CGB7* expression in metastatic urothelial tumors (data re-analyzed from Mariathasan et al., 2018). GSEA normalized Enrichment Score (NES): −2.20. FWER p-Value: 0.004. (**C**) Differentially expressed genes in *CGB7*+ vs *CGB7*− metastatic urothelial tumors (data re-analyzed from Mariathasan et al., 2018) as determined by Mann-Whitney U Test. Interferon gamma response genes that are significantly differentially expressed are in red (upregulated) or blue (downregulated) and reach p-value < 0.05 and log fold change > 1.5. CGB7+: >1 TPM. CGB7−: <0.25 TPM. (**D**) Differentially expressed genes in *CGB7*+ vs *CGB7*− primary urothelial tumors (data re-analyzed from TCGA) as determined by Mann-Whitney U Test. Interferon gamma response genes that are significantly differentially expressed are in red (upregulated) or blue (downregulated) and reach p-value < 0.05 and log fold change > 1.5. CGB7+: >1 TPM. CGB7−: <0.25 TPM. (**E**) Immunophenotype data in tumors stratified by *CGB7* expression: immune desert, immune excluded, or inflamed. P-values determined by proportions test with continuity correction. Chi-squared = 15.84, df = 1, p-value = 6.89×10^−5^. (**F**) As in (**E**), but stratified by *CGA* expression. Chi-squared = 0.88, df = 1, p-value = 0.35. (**G**) Tumor subtype data in tumors stratified by *CGB7* expression: basal/SCC-like, genomically unstable, immune infiltrated, urothelial type A, or urothelial type B. P-values determined by proportions test with continuity correction. Chi-squared = 30.95, df = 1, p-value = 2.65×10^−8^. (**H**) As in (**G**), but stratified by *CGA* expression. Chi-squared = 0.40, df = 1, p-value = 0.53. Med = medium.

As *CGB7* is the most frequently expressed of the CGB genes in our analyses of the TCGA urothelial carcinoma cohort, we elected to focus on *CGB7*. To interrogate the possible functional effects of *CGB7* expression in cancer, we re-analyzed RNA-sequencing data from the TCGA primary urothelial carcinoma cohort by comparing the gene expression profiles of *CGB7*-positive and *CGB7*-negative tumors. Gene Ontology (GO) enrichment analysis revealed that downregulated genes in *CGB7*-positive tumors are enriched for diverse biological processes (Figure S3A). Interestingly, one of the highest-ranked enriched GO terms was related to immune response, which we found to be particularly intriguing given previous literature demonstrating a functional role for hCG in immune tolerance in pregnancy (Schumacher et al., 2009; Schumacher et al., 2013; Poloskiet al., 2016; Dauven et al., 2016; Schumacher et al., 2017). Further probing of associated GO terms revealed that *CGB7* expression was specifically associated with downregulation of genes involved in the adaptive immune response, lymphocyte activation, and positive immune system regulation, indicating that *CGB7* expression is inversely associated with inflammation (Figure 3A) in a manner reminiscent of hCG’s documented role in maternal immunotolerance.

To confirm this finding in an orthogonal dataset, we analyzed mRNA-sequencing data from the IMVigor 210 multicentre, single-arm, phase II clinical trial (Mariathasan et al., 2018). This cohort is composed of patients with advanced urothelial carcinoma that received Atezolizumab, an anti-PD-L1 checkpoint inhibitor, and is ideally suited for our investigation given that pre-treatment samples were characterized by both RNA-seq and immunohistochemistry (Mariathasan et al., 2018). We again found that genes downregulated with *CGB7* expression were strongly associated with inflammatory processes (Figure 3A). Notably, in genes that are downregulated in *CGB7*-positive tumors, at least 17 of the 20 highest ranked enriched GO terms are robustly associated with inflammation and immunity, reinforcing a negative association between *CGB7* and inflammation (Figure S3B). The association between *CGB7* expression and regulation of immune response in this dataset is further supported by pronounced downregulation of genes involved in interferon gamma response in *CGB7*+ tumors identified by gene set enrichment analysis via GSEA software (Subramanian & Tamayo et al., 2005; Mootha & Lindgren et al., 2003). (Figure 3B).

Interferon gamma signaling is strongly associated with response to immune checkpoint inhibition, and indeed, disruption of interferon gamma signaling remains a mechanism of resistance to ICI (Zaretsky et al., 2016; Grasso et al., 2020). Exploring this *CGB7*-associated downregulation of interferon gamma response genes in both the IMVigor210 cohort dataset and in the TCGA urothelial carcinoma cohort dataset reveals downregulation of numerous genes encoding inflammatory cytokines and chemokines, immune checkpoints such as PD-L1, and HLA proteins involved in antigen presentation; all features of immune evasion in cancer (Figure 3C, 3D). As hCG is well-established as a facilitator of maternal immunosuppression (Schumacher et al., 2009; Schumacher et al., 2013; Poloski et al., 2016; Dauven et al., 2016; Schumacher et al., 2017), we hypothesize that *CGB7* may similarly exhibit immunosuppressive functions that ultimately promote immune escape of tumors.

As interferon gamma facilitates tumor infiltration of CD8+ T cells and is ultimately a marker of CD8+ T cell activity, we next investigated whether *CGB7* expression affects CD8+ T cell infiltration using immune phenotype classification data for tumor samples from the IMVigor 210 cohort. Here, immunophenotype is determined by CD8a IHC staining of FFPE tumor sections (Mariathasan et al., 2018). Tumors were scored on the basis of CD8a staining frequency and pattern as exhibiting an immune infiltrated, immune excluded, or immune desert phenotype (Mariathasan et al., 2018). We stratified tumors by *CGB7* expression and binned samples according to negative-low (<25%), moderate (25%−75%), or high (>75%) *CGB7* expression. Comparing the distribution of immunophenotypes between *CGB7*-low and *CGB7*-high tumors elucidates a significantly distinct distribution of inflamed tumors (p=6.89×10^−5^) (Figure 3E). We observed no statistically significant differences in the distributions of immune excluded or immune desert phenotypes between *CGB7*-low and *CGB7*-high tumors, though there is a trend toward higher incidence of immune exclusion in *CGB7*-high tumors (p= 0.062, p= 0.32). These findings that *CGB7*-high tumors exhibit decreased immune infiltration is consistent with our findings that *CGB7* expression is associated with downregulated IFNg signaling, as IFNg is predominantly secreted by activated lymphocytes, promotes tumor infiltration of immune cells such as CD8+ T cells, and is a marker of ICI response (Grasso et al., 2020).

Furthermore, when we compare *CGA*-low and *CGA*-high tumors, there is no significant difference in the distribution of inflamed tumors (p=0.35), supporting a possible *CGB7*-specific effect on CD8+ T cell exclusion (Figure 3F).

Additionally, when we explored associations between *CGB7* expression and subtype as scored by Mariathasan et al. according to the Lund classification system, an established system for urothelial cancer classification (Sjödahl et al., 2012; Sjödahl et al., 2013), we observe a significantly decreased fraction of immune-infiltrated tumors in *CGB7*-high tumors compared with *CGB7*-low tumors (p=2.65×10^−8^) (Figure 3G). This infiltrated class of tumors is described as enriched for immune system processes and lymphocyte activation, including an enrichment of activated T cell markers and cytotoxic T cell effector genes (Sjödahl et al., 2012). There was no association between the immune infiltrated subtype and *CGA* expression, further supporting an association between *CGB7* expression and reduced immune infiltration (p=0.53) (Figure 3H).

We focused these and prior analyses on *CGB7* because *CGB7* is more commonly expressed than other CGB genes in this cancer type (Figure 1E). Because some CGB-positive samples express only genes encoding type I CGBs (*CGB7*), others express only genes encoding type II CGBs *(CGB3, CGB5*, and *CGB8*), and others both type I and type II, we sought to test whether the functional association between CGB gene expression and immunophenotypes was likely driven by genes encoding type I or type II CGBs (or both). We used a patient stratification approach to address this question.

We first tested whether the associations between *CGB7* and CD8+ T cell infiltration and tumor subtype are upheld independently of *CGB3, CGB5*, and *CGB8* expression. We selected samples negative for *CGB3, CGB5*, and *CGB8* expression, and to ensure sufficient samples for analysis, we lowered the threshold for negative expression from 0.25 to 1 TPM to increase sample size. We then stratified samples by negative-low, moderate, or high *CGB7* expression as described. Consistent with our prior findings, *CGB7*-high tumors exhibit significantly decreased CD8+ T cell infiltration (incidence of inflamed tumors) compared with CGB7-low tumors (p=1.60×10–6) (Figure S3C). Similarly, *CGB7*-high tumors exhibit a significantly decreased fraction of immune infiltrated tumors compared with *CGB7*-low tumors (p=0.00450) (Figure S3D). Both of these findings are independent of *CGB3, CGB5*, and *CGB*8 expression. Conversely, selecting *CGB7*-negative samples and stratifying by summed *CGB3, CGB5*, and *CGB8* expression, we find no association between *CGB3*, *CGB5*, and *CGB8* expression and CD8+ T cell infiltration (p=0.730) or incidence of immune-infiltrated tumors (p=1) independent of *CGB7* expression (Figure S3E, S3F). It is important to note, however, that our statistical power for these analyses was not equal for type I and type II CGB analyses due to *CGB7* being the most commonly expressed CGB gene (Figure 1E).

Overall, these findings reinforce our hypothesis that *CGB7* is consistently associated with an immunosuppressive tumor microenvironment and hallmarks of immune escape. We conclude that *CGB7* is significantly associated with blunted interferon gamma signaling and is specifically and significantly associated with decreased CD8+T cell infiltration in the urothelial carcinoma cohorts investigated.

### CGB expression is associated with decreased response to ICI therapy

**Figure 4:**
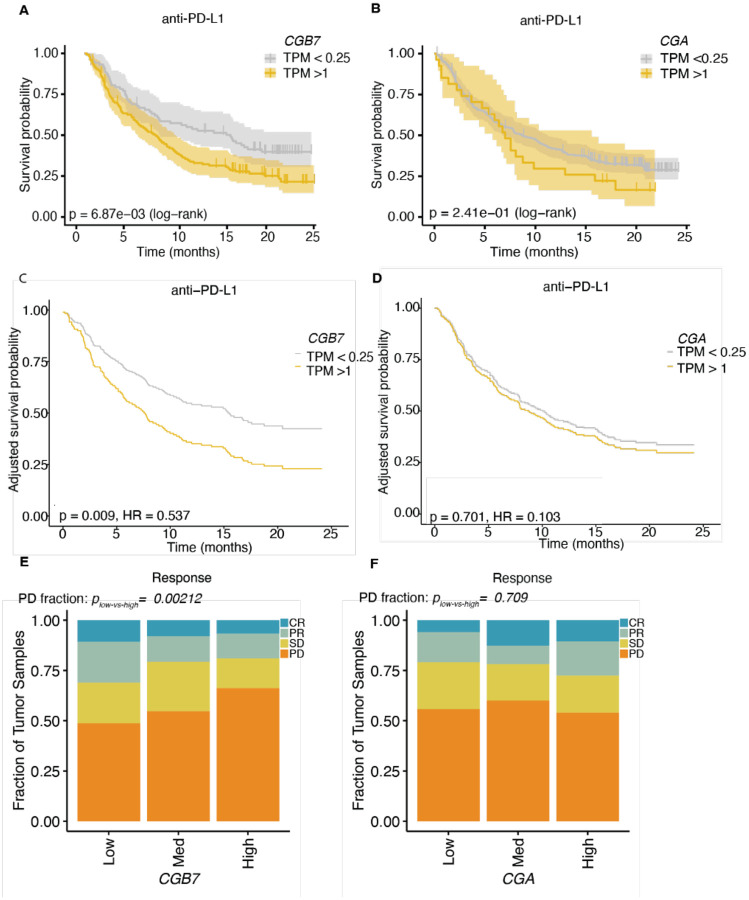
CGB expression is associated with decreased response to ICI therapy (**A**) Kaplan Meier overall survival curves comparing patients with *CGB7*+ (gold) and *CGB7*− (gray) advanced urothelial tumors undergoing treatment with Atezolizumab (anti-PD-L1). Data re-analyzed from Mariathasan et al., 2018. P-value determined by log rank test. The estimated survival functions (solid), censored events (crosses), and 95% confidence intervals (shaded regions) are shown. (**B**) As in (**A**), but patients stratified by *CGA* expression. (**C**) Kaplan Meier overall survival curves as shown in (**A**) adjusted for confounding effects of sex and tumor mutational burden covariates by Cox Proportional Hazards modeling. Hazard ratio (HR) and p-value obtained from fitting a Cox Proportional Hazards model. (**D**) Kaplan Meier overall survival curves as shown in (**B**) adjusted as described in (**C**). (**E**) Response determined by RECIST criteria (data re-analyzed from Balar et al., 2017). P-values determined by proportions test with continuity correction. Chi-squared = 9.44, df = 1, p-value = 0.0021. (**F**) As in (**A**), but stratified by *CGA* expression. Chi-squared = 0.14, df = 1, p-value = 0.71. Response Evaluation Criteria in Solid Tumors (RECIST) scoring: CR = complete response, PR = partial response, SD = stable disease, PD = progressive disease. Med = medium.

As interferon gamma release and CD8+ T cell infiltration are associated with response to ICI therapy, we next wondered whether *CGB7* expression had implications for overall survival and response to anti-PD-L1 checkpoint inhibition. To investigate this the effect of *CGB7* on survival, we selected *CGB7*-positive (TPM >1) and *CGB7*-negative (TPM < 0.25) tumors and performed Kaplan Meier survival analysis. We found that *CGB7* expression is indeed associated with significantly decreased overall survival probability (p= 6.87×10^−3^) (Figure 4A). Though this decrease in survival probability associated with *CGB7* expression is strongest, *CGB3* and *CGB5*, but not *CGB8*, are also associated with significantly decreased survival probability (Figure S4A–C). In contrast, *CGA* expression had no effect on overall survival (Figure 4B).

We wondered whether these associations between CGB gene expression and survival persist when controlling for common potential covariates tumor mutational burden (TMB) and sex. TMB is a known predictor of response to immune checkpoint inhibition (Samstein et al., 2019; Ricciuti et al., 2022; Hellmann et al., 2019; Hellmann et al., 2018). Sex is associated with differential survival outcomes in some checkpoint immunotherapy cohorts (Jang et al., 2021; Ye et al., 2020) and in this specific dataset (Figure S4I). Even when survival probabilities are adjusted for the contributions of TMB and sex using Cox proportional hazards modeling, the adjusted survival probability of patients with *CGB7*-positive cancers remains significantly decreased while *CGA* has no effect on adjusted overall survival probability (p= 0.009, p= 0.70) (Figure 4C, 4D). Similarly, the adjusted survival probabilities of patients with *CGB3*-positive and *CGB5*-positive tumors remains significantly decreased (Figure S4D–F). We conclude that *CGB3, CGB5*, and *CGB7* expression are associated with a significant reduction in survival probability in metastatic urothelial carcinoma patients receiving immune checkpoint blockade, with *CGB7* expression exhibiting the most robust association.

We next wondered whether *CGB7* expression impacts response to checkpoint immunotherapy. Each patient in the IMvigor 210 clinical trial was evaluated for response to anti-PD-L1 therapy by the Response Evaluation Criteria in Solid Tumors (RECIST) and scored as exhibiting progressive disease (PD), stable disease (SD), partial response (PR), or complete response (CR) (Balar et al., 2017). Again binning patients by *CGB7* expression corresponding to negative-low, moderate, or high expression levels, we compared the proportion of each clinical response classification in *CGB7*-high vs *CGB7*-low tumors to determine patterns of response associated with *CGB7* expression. *CGB7*-high tumors exhibited a significantly higher incidence of PD (p=0.0021), but no significant difference in incidence of CR (p = 0.25), PR (p = 0.051), or SD (p = 0.25) (Figure 4E). Conversely, comparing incidence of PD between *CGA*-high vs *CGA*-low tumors demonstrated no significant differences (p=0.71). Similarly, we observed no significant differences in incidence of CR (p = 0.074), PR (p =0.53), or SD (p = 0.12) between *CGA*-high vs *CGA*-low tumors (Figure 4F), highlighting a potential beta-subunit-specific effect on overall survival.

Repeating these analyses to parse *CGB7*-specific effects independent of *CGB3*, *CGB5*, and *CGB8* expression as described above, we determined that high *CGB7* expression is still associated with significantly increased incidence of progressive disease compared with low *CGB7* expression independently of *CGB3, CGB5*, and *CGB8* expression (p=0.0016) (Figure S4G). Interestingly, when *CGB7*-high samples are filtered out to facilitate bulk *CGB3, CGB5*, and *CGB8* analyses independent of *CGB7* expression, we observe that *CGB3, CGB5*, and *CGB8* are also significantly associated with increased incidence of progressive disease (p=0.0027) independently of *CGB7*, indicating that general CGB expression is associated with progressive disease and thus resistance to ICIs (Figure S4H).

Taken together, we conclude that *CGB7* expression is strongly associated with an immunosuppressive tumor microenvironment and decreased overall survival probability, suggesting a role of *CGB7* in immune escape of tumors. We additionally conclude that *CGB3* and *CGB5* are associated with decreased survival probability. Ultimately, expression of genes encoding both type I and type II CGB genes are associated with resistance to ICIs in advanced urothelial carcinoma

### CGB expression predicts response to ICI

We next sought to analyze the effect of *CGB7* expression on survival probability by further evaluating our Cox Proportional Hazards model. We first considered the effects of clinical variables collected in the IMVigor 210 trial, including *CGB7* expression, on overall survival using univariate Cox Proportional Hazards modeling, where variables are assessed separately, and in the multivariate setting, where confounding effects of all variables are controlled for (Figure S5A). In both approaches, tumor mutational burden (TMB), Eastern Cooperative Oncology Group (ECOG) status, and *CGB7* expression, which we treat as a continuous variable, were statistically significant prognostic variables associated with overall survival. Consistent with this notion, our analyses show that TMB is associated with a decreased risk of death (HR = 0.32, p = 1.10 × 10^−6^) after controlling for all other covariates. Similarly, ECOG status negatively impacts patient survival during ICI therapy as demonstrated in the original Phase II trial of the IMvigor210 study (Balar et al., 2017), which is reflected in our own analyses (ECOG 1: HR 2.62, p = 6.07 × 10^−8^; ECOG 2: HR 3.32, p = 2.30 × 10^−3^). Accordingly, increased *CGB7* expression is correlated with increased risk of death (HR = 1.48, p = 0.026) after controlling for possible confounding (Figure S5B).

**Figure 5:**
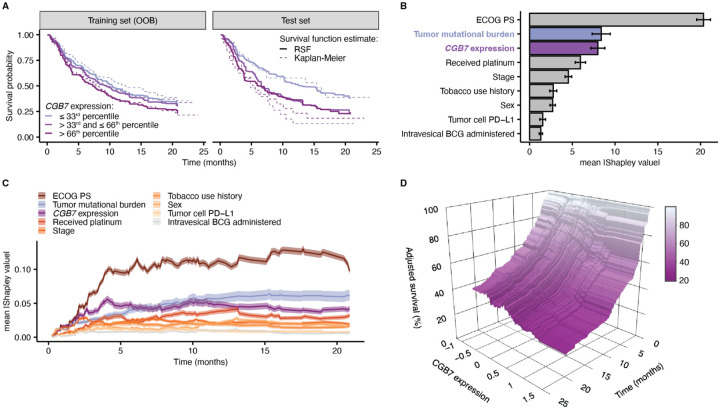
*CGB7* expression is a prognostic variable associated with decreased survival (**A**) Survival estimates from the Random Survival Forest (RSF) model (median prediction, solid lines) and the Kaplan–Meier estimate (dashed lines) from the training (OOB) and test sets. Patients are stratified into terciles based on *CGB7* expression. (**B**) RSF feature importance quantified via the mean absolute Shapley values. Error bars denote the 95% confidence intervals. (**C**) Time-dependent Shapley values, corresponding to survival estimates at specific timepoints. The 95% confidence interval of the mean (transparent ribbon) is shown. (**D**) Adjusted survival probability measured via partial dependence, as a function of time and *CGB7* expression.

We next aimed to quantify the predictive value of *CGB7* as a prognostic marker over time using inferences from a Random Survival Forest (RSF) model, a machine learning ensemble consisting of multiple survival trees (Ishwaran 2008). RSF modeling enables us to predict the time-to-death in our patient cohort, and has been used widely in the literature to predict the prognosis of patients in various disease and interventional contexts (Ishwaran & Malley, 2014; Pineda et al., 2024; Picket et al., 2021; Zhao et al., 2024; Sarica et al., 2023; Bohannan et al., 2022; Jung et al. 2019). To grow the RSF, we randomly selected 70% of the patients in the cohort, utilized 1000 base learners, and included the following covariates as input: ECOG status, TMB, *CGB7* expression, platinum chemotherapy history, stage, tobacco use, sex, tumor cell PD-L1 level, and intravesical BCG administration. The resultant model had an Out-of-Bag (OOB) error of 38.9%. The OOB error is calculated from predictions for unsampled patients from each bootstrapping iteration, and represents an unbiased estimate of the test error. We also directly measured model performance using the remaining 30% of patients excluded from model training, this test error measuring 35.6% (Figure S5B). The RSF survival curve estimates closely mirror those generated by Kaplan Meier survival analysis, lending confidence to our model (Figure 5A).

The survival probability predictions produced by the RSF model suggest that increasing *CGB7* expression is correlated with a decrease in median survival time, and conform with the aforementioned findings from the Cox Proportional Hazards regression (Fig 5A, Fig S5C). To quantify the contributions of *CGB7* expression to the predictions of time-to-death generated by the RSF model, we calculated the Shapley values corresponding to each variable utilized in our model (Lundberg & Lee, 2017; Maksymiuk et al., 2020; Shapley, 1953; Štrumbelj & Kononenko, 2014). Shapley values associated with the RSF prediction for mortality, the number of expected deaths over the observation window, were calculated which showed the expected protective effect of high TMB and the increased risk of death conferred by increased *CGB7* expression (Figure S5D–E). Ranking Shapley values by the mean magnitude calculated over the entire cohort allows quantification of a feature’s importance to the model prediction relative to the other model covariates. In these analyses, *CGB7* expression scored highly behind ECOG performance status and equivalently to TMB (Figure 5B). We extended these analyses to a time-dependent implementation. First, we verified the time frame upon which inferences from the RSF model are valid, through time-dependent Receiver Operating Characteristic (ROC) analyses. We calculated the cumulative/dynamic area under the ROC curve (AUC^C/D^) which quantifies the model’s accuracy at differentiating patient deaths occurring before a particular time point, versus those who survive beyond this time. Our analyses suggest a time horizon up to ~ 15 months (Figure S5F). Second, we calculated Shapley values associated with time point-specific survival probability, which suggest that *CGB7* expression has a dynamic relative contribution over time but preserved high importance throughout the observation window. Interestingly, *CGB7* expression is linked to greater prediction contributions compared to TMB at earlier times, up to ~ 6 months (Figure 5C). To obtain the adjusted or marginal effects of *CGB7* expression on overall survival, we implemented time-dependent partial dependence analyses which show a clear negative correlation between *CGB7* expression and survival probability (Figure 5D). Ultimately, we observe a robust association between *CGB7* expression and predicted survival probability, in which *CGB7* expression is associated with reduced time to death in advanced urothelial cancer in the context of immune checkpoint inhibition.

## DISCUSSION

We identify CGB genes as biomarkers of immune evasion in cancer and as prognostic factors for response to immune checkpoint inhibition. We find that all CGB genes are extensively expressed across cancer types, with *CGB7* being most frequently expressed. We confirm that CGB genes are expressed and the resultant proteins secreted from urothelial carcinoma cells *in vitro*. In a cohort of advanced urothelial carcinoma patients, *CGB7* expression is associated with reduced CD8+ T cell infiltration. *CGB7* and bulk expression of *CGB3*, *CGB5*, and *CGB8* are associated with reduced response to anti-PD-L1 checkpoint immunotherapy by RECIST criteria. We subsequently identify that tumors expressing *CGB7, CGB3*, or *CGB5* are significantly associated with decreased survival probability via Kaplan-Meier survival analyses, and this association is upheld with correction for TMB and sex via Cox Proportional Hazards modeling. As *CGB7* expression is most frequently observed in urothelial carcinoma and is associated with markers of immune evasion, we focused on investigating the predictive value of *CGB7* and ultimately demonstrate the prognostic value of *CGB7* as a marker of poor prognosis in urothelial carcinoma in the context of immune checkpoint inhibition using Random Survival Forest modeling. In fact, *CGB7* is the second most important variable in survival predictions, behind TMB, early in cancer progression. Taken together, our data suggest that *CGB7* may facilitate the conversion of the tumor microenvironment to an immunosuppressive state.

One limitation of this study is the lack of functional characterization of CGB proteins in cancer, as functional immunology efforts are made challenging by the primate-specific nature of CGB genes and lack of murine orthologs. These functional studies, however, will be necessary to establish the immunosuppressive mechanism of action of CGB7. Our findings here demonstrate that, with careful experimental design and immune competent models, the study of CGB7 as an immunomodulator is ripe for further functional exploration. The primate-specificity of CGB gene expression further reinforces the value of analyzing high quality patient data, and the value of extending our analyses of *CGB7* to additional patient datasets.

A second limitation is the limited power of our current analyses dissecting isolated phenotypic associations with *CGB7* expression and with bulk summed *CGB3, CGB5*, and *CGB8* expression due to study size. In the analyses that we present here, it is interesting that, while *CGB7* is the only CGB gene associated with markers of immune evasion, multiple CGB genes are associated with ICI resistance and decreased survival. This might reflect a unique mechanism of action of *CGB7*, or perhaps suggest that the potential immunosuppressive functions of CGB genes are only one part of the mechanism underlying their association with poor prognosis. For example, hCG facilitates many pro-decidualization and pro-tumorigenic processes such as invasion, angiogenesis, in addition to immunosuppression. Our preliminary analyses suggest that these associations with *CGB7* are independent of *CGB3, CGB5* and *CGB8. CGB7* and bulk *CGB3, CGB5, and CGB8* are each independently associated with ICI resistance. Additional analyses to tease apart the isolated contributions of each gene will be required, and larger cohorts and study sizes will facilitate this. We hypothesize that, based on our findings in this study, while *CGB7* may uniquely be a marker of immune suppression and has great value as a marker or poor prognosis based on our analyses, that expression of any CGB gene has value as a marker of ICI resistance and worsened survival probability.

As primary and acquired resistance to checkpoint inhibitor immunotherapies remains a significant barrier to long-term remission for the majority of patients, further validation of *CGB7* as a biomarker and characterization of the mechanism by which *CGB7* suppresses the anti-cancer effects of ICIs may help us predict patient response, inform treatment, and perhaps even identify CGB7 as a potential therapeutic target. As CGB proteins are predominantly cancer-specific and readily detectable analytes in urine and plasma as the basis of modern pregnancy tests, this highlights the feasibility of CGB as a cancer biomarker. Further work will be required to validate CGB7 as a pan-cancer biomarker by confirming associations between *CGB7* expression and immunosuppressive signatures in other cancer types, and ultimately by validating this association between *CGB7* expression and ICI response in patient samples.

Ultimately, targeting CGB genes could present a promising approach for combination therapy with immunotherapies such as immune checkpoint inhibitors. As CGB proteins are secreted, there is substantial potential for development of an inhibitory anti-pan-CGB or anti-CGB7 antibody. There is precedent for potential therapeutic value of antibody neutralization of CGB proteins in the form of anti-cancer vaccines (Moulton 2002), and an anti-CGB therapeutic antibody could potentially provide therapeutic value on its own, or to provide specificity to a coupled anti-cancer payload.

## METHODS

### Datasets

**Table:** 

Dataset name	Accession number(s)	PMID(s)
TCGA BLCA	NCI Genomic Data Commons	
TCGA BRCA	NCI Genomic Data Commons	
TCGA CESC	NCI Genomic Data Commons	
TCGA CHOL	NCI Genomic Data Commons	
TCGA COAD	NCI Genomic Data Commons	
TCGA ESCA	NCI Genomic Data Commons	
TCGA GBM	NCI Genomic Data Commons	
TCGA HNSC	NCI Genomic Data Commons	
TCGA KICH	NCI Genomic Data Commons	
TCGA KIRC	NCI Genomic Data Commons	
TCGA KIRP	NCI Genomic Data Commons	
TCGA LIHC	NCI Genomic Data Commons	
TCGA LUAD	NCI Genomic Data Commons	
TCGA LUSC	NCI Genomic Data Commons	
TCGA PAAD	NCI Genomic Data Commons	
TCGA PCPG	NCI Genomic Data Commons	
TCGA PRAD	NCI Genomic Data Commons	
TCGA STAD	NCI Genomic Data Commons	
TCGA THYM	NCI Genomic Data Commons	
TCGA UCEC	NCI Genomic Data Commons	
Mariathasan et al., 2018	EGAD00001003977 (EGA)	29443960

### Cell lines

All cell lines utilized are human urothelial cancer cell lines cultured at 37°C and 5% CO2. SCaBER, T24, TCCSUP, J82, UM-UC-3, cell lines were purchased from ATCC (ATCC, Manassas, VA). RT-112 and KU-19–19 cell lines were purchased from DSMZ (DSMZ, Braunschweig, Germany). UM-UC-1 cell line was purchased from Sigma-Aldrich (St. Louis, MO). SCaBER, RT-112, UM-UC-1, UM-UC-3, TCCSUP and J82 cells were cultured in Eagle’s Minimum Essential Medium (ATCC) supplemented with 10% FBS (Gibco, Waltham, MA) and 1% Pen Strep (Gibco). T24 cells were cultured in McCoy’s 5A medium supplemented with 10% FBS and 1% Pen Strep. KU-19–19 cells were cultured in RPMI 1640 (Gibco) medium supplemented with 10% FBS and 1% Pen Strep.

### CGB knockdown experiments

siRNA pools targeted all CGB genes (Horizon Discovery, Cambridge, Cambridgeshire, UK) or were a non-targeting control pool (Horizon Discovery). siRNAs transfected into cells of approximately 70% confluence with Lipofectamine^™^ RNAiMAX Transfection Reagent (Thermo Fisher Scientific, Waltham, MA) per the manufacturer’s instructions.

### ELISA

Conditioned medium: conditioned medium was collected from each indicated cell line after 72 hours of subculture in the medium described above. Bulk beta-hCG protein levels were measured and quantified using a Human CG beta (HCG beta) DuoSet ELISA kit (R&D Systems, Minneapolis, MN) DuoSet ELISA Ancillary Reagent Kit 2 (R&D Systems) according to the manufacturer’s instructions. Media was diluted 1:10 from all cell lines with the exception of KU-19–19, which was diluted 1:100 prior to assay input due to high CGB protein levels. Cells were counted at collection to approximate concentration of CGB per 10,000 cells. Experiments were run in biological and technical triplicate. Raw O.D. values of standards were fit to a 4PL curve using MyAssays software.

### qRT-PCR

Total RNA was extracted using a Qiagen RNeasy plus mini kit according to the manufacturer’s instructions (Qiagen, Hilden, Germany). RNA was reverse transcribed to cDNA using a Verso cDNA synthesis kit (Thermo Scientific). *CGB7* transcripts detected with Applied Biosystems TaqMan Fast Advanced Master Mix (Fisher Scientific), *CGB7*-specific Taqman Probe (Thermo Scientific), *CGB3*/*CGB5*/*CGB8*-specific Taqman Probe (Thermo Scientific), and GAPDH Taqman Probe (Thermo Scientific). Experiments were run in biological and technical triplicate. Experiments were run on a ABI QuantStudio 5 Real-Time PCR System (Thermo Fisher Scientific). Data was analyzed using the ΔΔCt method.

### RNA sequencing analysis, genome annotations and differential gene expression

RNA-sequencing data was analyzed as previously described (Pineda 2024). RNA-seq reads were mapped to an annotated transcriptome created using Ensembl 71 (Flicek et al., 2013), UCSC knownGene (Meyer et al., 2013), and MISO v2.0 (Katz et al., 2010) annotations for the hg19/GRCh37 assembly using RSEM version 1.2.4 (B. Li & Dewey, 2011) modified to call Bowtie v1.0.0 with option ‘-v 2’ (Langmead et al., 2009). Unaligned reads were then mapped to the hg19/GRCh37 genome assembly using TopHat version 20.8b (Trapnell et al., 2009). Gene expression values in TPM (transcripts per million) were normalized via the trimmed mean of M values (TMM) method (M. D. Robinson & Oshlack, 2010). For TCGA studies, we analyzed all available samples across 20 distinct cancer types that included at least one matched non-tumor tissue sample. Differential gene expression to compare positive and negative samples for expression of CGB genes in both the TCGA datasets and the dataset from the IMvigor210 clinical trial cohort (Mariathasan et al. 2018, Balar et al., 2017) was calculated via Mann-Whitney U Test and using several thresholds: the absolute value of log-fold-change threshold of 1.5 or greater, a maximum p-value of 0.05, a minimum bayes factor of 100, and maximum FDR of 0.01. We defined positive expression of CGB genes as TPM >1 and negative expression as TPM < 0.25 unless otherwise noted.

### Clinical variable analyses

Clinical variables were reported for patients enrolled in the IMVigor210 clinical trial (Mariathasan et al., 2018, Balar et al., 2017). Patient response to Atezolizumab was scored according to the Response Evaluation Criteria for Solid Tumors (RECIST) (Balar et al., 2017). Tumor subtype was determined according to Lund 2 taxonomy for bladder cancer classification (Mariathasan 2018). Immunophenotype was determined based on the level and pattern of CD8+ T cell infiltration by CD8a IHC staining (Mariathasan et al., 2018). For each analysis, patients were first stratified by expression of each CGB gene, or by summed expression of multiple CGBs where indicated. NAs were filtered out for each response, immunophenotype, and subtype analysis individually after CGB gene stratification. We determined the proportions of each RECIST score, immunophenotype, and subtype in CGB-high (>75%) and CGB-low/negative (<25%) tumors, and quantified the difference in proportions via 1-sample proportions test with continuity correction.

### Survival analyses

Survival analyses were performed with the Kaplan–Meier estimator and statistical tests were performed with a logrank test (R package survival) (Therneau 2024). Samples were stratified by expression of individual genes, where positive expression was defined as exhibiting TPM >1, and negative expression was defined as exhibiting TPM <0.25. Adjusted survival probabilities were calculated by fitting a Cox Proportional Hazards model to adjust for the covariates sex and tumor mutational burden.

### Random Survival Forest and Feature Importance computation

The Random Survival Forest Model, Shapley value estimation, and partial dependence analyses was implemented as previously described (Pineda 2024). In brief, we randomly assigned patients into training (70%) and test (30%) datasets. We determined optimal hyperparameters via a grid search. That is, we evaluated 10,608 RSF models representing various values and combinations of the RSF hyperparameters: number of trees, minimum terminal node size, number of randomly selected splitting variables, handling of missing data, splitting rule, and bootstrapping method. The model which minimized both the OOB training and the test errors (defined as 1 − concordance index) was selected: ntree = 1000, nodesize = 6, mtry = 3, na.action = “na.impute”, splitrule = “logrank”, and samptype = “swr”. We set the hyperparameter nsplit = 0 to evaluate all possible split points. The predictions associated with the test cohort were handled using na.action = “na.omit” which excluded patients with missing data. Shapley values were calculated using the fastshap package (Greenwell, 2024). We used 1000 Monte Carlo repetitions and set the parameter adjust = TRUE to correct the estimated Shapley values such that local accuracy was satisfied. Shapley values associated with mortality and per time point overall survival predictions from the RSF model were used to quantify overall feature importance and time-dependent importance, respectively. The marginal effect of CGB7 expression on survival probability was assessed using partial dependence, implemented using the partial() function from the randomForestSRC package. Visualizations were created in the R programming environment using the dplyr (Wickham et al., 2022), ggplot2 (Wickham, 2016), pammtools (Bender and Scheipl, 2018), and plotly (Sievert, 2020) packages.

### Measuring survival model predictive accuracy

We evaluated the RSF model’s accuracy over time using time-dependent ROC curve analyses. For each timepoint, we calculated the cumulative/dynamic area under the ROC curve (AUC^C/D^) and 95% confidence interval using the timeROC package, which additionally corrects for bias due to right-censoring (Blanche et al., 2013). The training (OOB) or the test predictions for mortality were used as input.

## Supplementary Material

1

## Figures and Tables

**Table 1:** Expression of Chorionic Gonadotropin genes in cell lines from the Cancer Cell Line Encyclopedia

Cell Line	*CGB3*	*CGB5*	*CGB7*	*CGB8*	*CGA*	Sum of CGB genes
KU-19-19	0.56	229.23	2.45	14.12	0.00	246.35
J82	2.50	25.04	4.17	36.35	0.75	68.06
UM-UC-3	0.36	0.71	0.41	28.27	0.00	29.76
SCaBER	1.42	2.63	2.20	16.69	0.45	22.93
RT-112	0.28	3.11	3.79	10.81	0.33	18.00
UM-UC-1	0.25	2.19	1.81	11.97	0.42	16.22
639-V	0.75	7.79	1.66	3.78	0.26	13.98
VM-CUBl	0.50	3.23	7.22	1.81	0.00	12.75
JMSU-1	0.14	1.36	1.33	4.37	0.23	7.19
5637	0.11	1.41	2.10	2.63	0.39	6.25
HT-1197	0.62	0.45	2.45	1.98	1.06	5.50
SW-1710	0.12	0.15	1.95	2.74	0.32	4.96
KMBC-2	0.10	1.56	1.93	1.35	0.76	4.95
647-V	0.11	1.22	0.37	1.89	0.00	3.59
CAL-29	0.18	1.29	1.04	0.64	0.42	3.14
HT-1376	0.38	0.74	1.00	0.29	9.73	2.41
RT4	0.08	0.33	1.91	0.05	0.22	2.38
BFTC-905	0.10	0.41	1.46	0.14	0.32	2.11
BC-3C	0.11	0.21	0.11	0.79	0.34	1.22
T24	0.05	0.09	0.53	0.05	0.00	0.72
TCCSUP	0.06	0.08	0.39	0.08	0.27	0.62

Values in Transcripts Per Million (TPM).

**Table 2:** Cox Proportional Hazards Regression for Overall Survival

	Univariate				Multivariate^a,b^			
Characteristic	HR	95% CI	p-value	q-value^c^	HR	95%CI	p-value	q-value^c^
Tumor mutational burden^d^	0.43	0.28 – 0.66	1.10 × 10–4	7.72 × 10–4	0.32	0.19 – 0.50	1.10 × 10–6	7.73 × 10–6
*CGB7* expression^e^	1.44	1.09 – 1.89	9.05 × 10–3	0.04	1.48	1.05 – 2.11	0.026	0.093
ECOG Performance Status^f^:								
0 (reference)	-	-	-	-	-	-	-	-
1	2.1	1.58 – 2.79	2.95 × 10–7	4.14 × 10–6	2.62	1.85 – 3.71	6.07 × 10–8	8.50 × 10–7
2	1.97	1.04 – 3.73	0.036	0.13	3.32	1.53 – 7.17	2.30 × 10–3	0.011
Received platinum:								
No (reference)	-	-	-	-	-	-	-	-
Yes	1.41	1.01 – 1.98	0.047	0.13	1.54	1.03 – 2.30	0.037	0.1
Tumor cell PD-L1^g^:								
< 1% (reference)	-	-	-	-	-	-	-	-
≥ 1% but < 50%	1.12	0.66 – 1.90	0.66	0.99	1.14	0.64 – 2.02	0.65	0.83
≥ 50%	1	0.70 – 1.44	0.99	0.99	0.77	0.49 – 1.23	0.27	0.54
Intravesical BCG administered:								
No (reference)	-	-	-	-	-	-	-	-
Yes	0.98	0.73 – 1.32	0.89	0.99	0.92	0.63 – 1.33	0.65	0.83
Tobacco use history:								
Never (reference)	-	-	-	-	-	-	-	-
Previous	0.9	0.68 – 1.19	0.45	0.78	1.01	0.72 – 1.42	0.95	0.95
Current	1.02	0.65 – 1.61	0.92	0.99	1.09	0.63 – 1.90	0.75	0.87
Stage:								
I (reference)	-	-	-	-	-	-	-	-
II	0.96	0.69 – 1.35	0.83	0.99	1.25	0.80 – 1.95	0.33	0.57
III	1.23	0.86 – 1.75	0.25	0.51	1.4	0.90 – 2.19	0.14	0.32
IV	0.98	0.68 – 1.42	0.92	0.99	0.89	0.54 – 1.46	0.65	0.83
Sex:								
Male (reference)	-	-	-	-	-	-	-	-
Female	1.23	0.91 – 1.66	0.18	0.42	1.05	0.72 – 1.53	0.81	0.87

a Log-rank test: p-value = 1.05 × 10–7

b Akaike Information Criterion =1736.51; Bayesian Information Criterion = 1780.66; Harrell’s Concordance Index = 0.67

c Benjamini-Hochberg FDR correction

d log_10_(number of missense mutations)

e log_10_[CGB7 expression (TPM)]

f Eastern Oncology Cooperative Group Performance Status

g Fraction of tumor cells with positive PD-L1 staining

**Table:** Key Resources Table

Reagent type or resource	Designation	Source or reference	Identifiers	Additional information
Cell line (human)	T24	ATCC	HTB-4	
Cell line (human)	TCCSUP	ATCC	HTB-5	
Cell line (human)	SCaBER	ATCC	HTB-3	
Cell line (human)	J82	ATCC	HTB-1	
Cell line (human)	UM-UC-3	ATCC	CRL-1749	
Cell line (human)	UM-UC-1	Sigma-Aldrich	06080301-1VL	
Cell line (human)	RT-112	DSMZ	ACC 418	
Cell line (human)	KU-19-19	DSMZ	ACC 395	
sequence-based reagent	siRNA pool: targeting CGB genes	Horizon Discovery	L-01116400-0005	
sequenced-based reagent	siRNA pool: nontargeting control	Horizon Discovery	D-00181010-05	
Commercial assay, kit	Lipofectamine^™^ RNAiMAX Transfection Reagent	Thermo Fisher Scientific	13778150	
Commercial assay, kit	Human CG beta (HCG beta) DuoSet ELISA kit	R&D Systems	DY9034-05	
Commercial assay, kit	DuoSet ELISA Ancillary Reagent Kit 2	R&D Systems	DY008B	
Commercial assay, kit	RNeasy plus mini kit	Qiagen	74134	
Commercial assay, kit	Verso cDNA synthesis kit	Thermo Scientific	AB1453B	
Commercial assay, kit	Applied Biosystems TaqMan Fast Advanced Master Mix	Fisher Scientific	44-445-57	
Commercial assay, kit	CGB7-specific Taqman Probe	Thermo Scientific	Hs01668436_g1	
Commercial assay, kit	CGB3/CGB5/CGB8-specific Taqman Probe	Thermo Scientific	Hs03407508_gH	
Commercial assay, kit	GAPDH Taqman Probe	Thermo Scientific	Hs02786624_g1	
Software, algorithm	UCSC knownGene	Meyer et al., 2013; PMID:23155063		
Software, algorithm	Ensembl 71	Flicek et al., 2013; PMID:23203987		
Software, algorithm	MISO v2.0	Katz et al., 2010; PMID:21057496		
Software, algorithm	RSEM v1.2.4	Li and Dewey, 2011; PMID:21816040		
Software, algorithm	Bowtie v1.0.0	Langmead et al., 2009; PMID:19261174		
Software, algorithm	TopHat v.2.0.8b	Trapnell et al., 2009; PMID:19289445		
Software, algorithm	Trimmed mean of *M* values (TMM) method	Robinson and Oshlack, 2010; PMID:20196867		
Software, algorithm	clusterProfiler	Wu et al., 2021; Yu et al., 2012; PMID:34557778, 22455463		
Software, algorithm	samtools	Li et al., 2009; PMID:19505943		
Software, algorithm	kallisto v.0.46.1	Bray et al., 2016; PMID:27043002		
Software, algorithm	survival	Therneau and Grambsch, 2000; Therneau, 2022; https://github.com/therneau/survival		
Software, algorithm	stats	R Development Core Team, 2022; https://www.r-project.org/		
Software, algorithm	ggplot2	Wickham, 2016; https://github.com/tidyverse/ggplot2		
Software, algorithm	dplyr	Wickham et al., 2022; https://github.com/tidyverse/dplyr		
Software, algorithm	survminer	Kassambara et al., 2021; https://rpkgs.datanovia.com/survminer/index.html		
Software, algorithm	randomForestS RC	Ishwaran et al., 2008; https://www.randomforestsrc.org/articles/survival.html		
Software, algorithm	fastshap	Greenwell, 2021; https://github.com/bgreenwell/fastshap		
Software, algorithm	pammtools	Bender and Scheipl, 2018; https://adibender.github.io/pammtools/		
Software, algorithm	plotly	Sievert, 2020; https://plotly.com/r/		
Software, algorithm	timeROC	Blanche et al., 2013; https://CRAN.R-project.org/package=timeROC		
Software, algorithm	MyAssays	MyAssays (RRID:SCR_016562)		

## References

[1] Lázaro-Gorines R., Ruiz-de-la-Herrán J., Navarro R. A novel Carcinoembryonic Antigen (CEA)-Targeted Trimeric Immunotoxin shows significantly enhanced Antitumor Activity in Human Colorectal Cancer Xenografts. Sci Rep 9, 11680 (2019). 10.1038/s41598-019-48285-z 31406218 PMC6690998

[2] Terentiev AA, Moldogazieva NT. Alpha-fetoprotein: a renaissance. Tumour Biol. 2013 Aug;34(4):2075–91. doi: 10.1007/s13277-013-0904-y. Epub 2013 Jun 14. 23765762

[3] Sandberg ML, Wang X, Martin AD, Nampe DP, Gabrelow GB, Li CZ, McElvain ME, Lee WH, Shafaattalab S, Martire S, Fisher FA, Ando Y, Liu E, Ju D, Wong LM, Xu H, Kamb A. A carcinoembryonic antigen-specific cell therapy selectively targets tumor cells with HLA loss of heterozygosity in vitro and in vivo. Sci Transl Med. 2022 Mar 2;14(634):eabm0306. doi: 10.1126/scitranslmed.abm0306. Epub 2022 Mar 2. 35235342

[4] Munson PV, Adamik J, Butterfield LH. Immunomodulatory impact of α-fetoprotein. Trends Immunol. 2022 Jun;43(6):438–448. doi: 10.1016/j.it.2022.04.001. Epub 2022 May 9. 35550875

[5] Lapthorn AJ, Harris DC, Littlejohn A, Lustbader JW, Canfield RE, Machin KJ, Morgan FJ, Isaacs NW. Crystal structure of human chorionic gonadotropin. Nature. 1994 Jun 9;369(6480):455–61. doi: 10.1038/369455a0. 8202136

[6] Douglas J. Serum total hCGβ level is an independent prognostic factor in transitional cell carcinoma of the urothelial tract. Brit J Cancer 110, 1759–1766 (2014). 24556622 10.1038/bjc.2014.89 PMC3974095

[7] Iles RK. Ectopic hCGbeta expression by epithelial cancer: malignant behaviour, metastasis and inhibition of tumor cell apoptosis. Mol Cell Endocrinol. 2007 Jan 2;260–262:264–70. doi: 10.1016/j.mce.2006.02.019. Epub 2006 Oct 27. 17069968

[8] Chew GL, Campbell AE, De Neef E, Sutliff NA, Shadle SC, Tapscott SJ, Bradley RK. DUX4 Suppresses MHC Class I to Promote Cancer Immune Evasion and Resistance to Checkpoint Blockade. Dev Cell. 2019 Sep 9;50(5):658–671.e7. doi: 10.1016/j.devcel.2019.06.011. Epub 2019 Jul 18. 31327741 PMC6736738

[9] Chang C, Chen YL, Wang YW, Chen HW, Hsu CW, Lin KC, Ou YC, Liu T, Chen WL, Chu CA, Ho CL, Lee CT, Chow NH. Aberrant trophoblastic differentiation in human cancer: An emerging novel therapeutic target (Review). Oncol Rep. 2024 Mar;51(3):43. doi: 10.3892/or.2024.8701. Epub 2024 Jan 19. 38240107 PMC10823338

[10] Cheng HL, Chou LP, Tsai HW, Lee CT, Wang YW, Chung-Liang H, Ou JH, Tsai YS, Chow NH. Urothelial carcinoma with trophoblastic differentiation: Reappraisal of the clinical implication and immunohistochemically features. Urol Oncol. 2021 Oct;39(10):732.e17–732.e23. doi: 10.1016/j.urolonc.2021.03.006. Epub 2021 Mar 26. 33773916

[11] Fluhr H, Bischof-Islami D, Krenzer S, Licht P, Bischof P, Zygmunt M. Human chorionic gonadotropin stimulates matrix metalloproteinases-2 and −9 in cytotrophoblastic cells and decreases tissue inhibitor of metalloproteinases-1, −2, and −3 in decidualized endometrial stromal cells. Fertil Steril. 2008 Oct;90(4 Suppl):1390–5. doi: 10.1016/j.fertnstert.2007.08.023. Epub 2008 Mar 4. 18291374

[12] Guibourdenche J., Handschuh K., Tsatsaris V., Gerbaud P., Leguy M. C., Muller F., Evain Brion D., Fournier T., Hyperglycosylated hCG Is a Marker of Early Human Trophoblast Invasion, The Journal of Clinical Endocrinology & Metabolism, Volume 95, Issue 10, 1 October 2010, Pages E240–E244, 10.1210/jc.2010-0138 20660042

[13] Zygmunt M, Herr F, Keller-Schoenwetter S, Kunzi-Rapp K, Münstedt K, Rao CV, Lang U, Preissner KT. Characterization of human chorionic gonadotropin as a novel angiogenic factor. J Clin Endocrinol Metab. 2002 Nov;87(11):5290–6. doi: 10.1210/jc.2002-020642. 12414904

[14] Berndt S, Perrier d’Hauterive S, Blacher S, Péqueux C, Lorquet S, Munaut C, Applanat M, Hervé MA, Lamandé N, Corvol P, van den Brûle F, Frankenne F, Poutanen M, Huhtaniemi I, Geenen V, Noël A, Foidart JM. Angiogenic activity of human chorionic gonadotropin through LH receptor activation on endothelial and epithelial cells of the endometrium. FASEB J. 2006 Dec;20(14):2630–2. doi: 10.1096/fj.06-5885fje. Epub 2006 Oct 25. 17065221

[15] Berndt S, Blacher S, Perrier d’Hauterive S, Thiry M, Tsampalas M, Cruz A, Péqueux C, Lorquet S, Munaut C, Noël A, Foidart JM. Chorionic gonadotropin stimulation of angiogenesis and pericyte recruitment. J Clin Endocrinol Metab. 2009 Nov;94(11):456–774. doi: 10.1210/jc.2009-0443. Epub 2009 Oct 16. 19837939

[16] Schumacher A, Brachwitz N, Sohr S, Engeland K, Langwisch S, Dolaptchieva M, Alexander T, Taran A, Malfertheiner SF, Costa SD, Zimmermann G, Nitschke C, Volk HD, Alexander H, Gunzer M, Zenclussen AC. Human chorionic gonadotropin attracts regulatory T cells into the fetal-maternal interface during early human pregnancy. J Immunol. 2009 May 1;182(9):5488–97. doi: 10.4049/jimmunol.0803177. 19380797

[17] Schumacher A, Heinze K, Witte J, Poloski E, Linzke N, Woidacki K, Zenclussen AC. Human chorionic gonadotropin as a central regulator of pregnancy immune tolerance. J Immunol. 2013 Mar 15;190(6):2650–8. doi: 10.4049/jimmunol.1202698. Epub 2013 Feb 8. 23396945

[18] Dauven D, Ehrentraut S, Langwisch S, Zenclussen AC, Schumacher A. Immune Modulatory Effects of Human Chorionic Gonadotropin on Dendritic Cells Supporting Fetal Survival in Murine Pregnancy. Front Endocrinol (Lausanne). 2016 Nov 15;7:146. doi: 10.3389/fendo.2016.00146. P 27895621 PMC5108759

[19] Poloski E, Oettel A, Ehrentraut S, Luley L, Costa SD, Zenclussen AC, Schumacher A. JEG-3 Trophoblast Cells Producing Human Chorionic Gonadotropin Promote Conversion of Human CD4+FOXP3− T Cells into CD4+FOXP3+ Regulatory T Cells and Foster T Cell Suppressive Activity. Biol Reprod. 2016 May 1;94(5):106. doi: 10.1095/biolreprod.115.135541. Epub 2016 Mar 9. 26962115

[20] Schumacher A. Human Chorionic Gonadotropin as a Pivotal Endocrine Immune Regulator Initiating and Preserving Fetal Tolerance. Int J Mol Sci. 2017 Oct 17;18(10):2166. doi: 10.3390/ijms18102166. 29039764 PMC5666847

[21] Bellet D, Lazar V, Bieche I, Paradis V, Giovangrandi Y, Paterlini P, Lidereau R, Bedossa P, Bidart J-M, Vidaud M. Malignant transformation of nontrophoblastic cells is associated with the expression of chorionic gonadotropin beta genes normally transcribed in trophoblastic cells. Cancer Res 1997; 57:516–523. 9012484

[22] Sohr S, Engeland K. The tumor suppressor p53 induces expression of the pregnancy-supporting human chorionic gonadotropin (hCG) CGB7 gene. Cell Cycle. 2011 Nov 1;10(21):3758–67. doi: 10.4161/cc.10.21.17946. Epub 2011 Nov 1. 22032922 PMC3266010

[23] Zimmermann G, Ackermann W, Alexander H. Expression and production of human chorionic gonadotropin (hCG) in the normal secretory endometrium: evidence of CGB7 and/or CGB6 beta hCG subunit gene expression. Biol Reprod. 2012 Mar 30;86(3):87. doi: 10.1095/biolreprod.111.092429. 21832169

[24] Subramanian A, Tamayo P, Mootha VK, Mukherjee S, Ebert BL, Gillette MA, Paulovich A, Pomeroy SL, Golub TR, Lander ES, Mesirov JP. Gene set enrichment analysis: a knowledge-based approach for interpreting genome-wide expression profiles. Proc Natl Acad Sci U S A. 2005 Oct 25;102(43):15545–50. doi: 10.1073/pnas.0506580102. Epub 2005 Sep 30. 16199517 PMC1239896

[25] Subramanian A, Tamayo P, Mootha VK, Mukherjee S, Ebert BL, Gillette MA, Paulovich A, Pomeroy SL, Golub TR, Lander ES, Mesirov JP. Gene set enrichment analysis: a knowledge-based approach for interpreting genome-wide expression profiles. Proc Natl Acad Sci U S A. 2005 Oct 25;102(43):15545–50. doi: 10.1073/pnas.0506580102. Epub 2005 Sep 30. 16199517 PMC1239896

[26] Cook AM, Huddart RA, Jay G, Norman A, Dearnaley DP, Horwich A. The utility of tumour markers in assessing the response to chemotherapy in advanced bladder cancer. Br J Cancer. 2000 Jun;82(12):1952–7. doi: 10.1054/bjoc.2000.1147. 10864203 PMC2363245

[27] Hotakainen K, Haglund C, Paju A, Nordling S, Alfthan H, Rintala E, Stenman UH. Chorionic gonadotropin beta-subunit and core fragment in bladder cancer: mRNA and protein expression in urine, serum and tissue. Eur Urol. 2002 Jun;41(6):677–85. doi: 10.1016/s0302-2838(02)00125-2. 12074787

[28] Moutzouris G, Yannopoulos D, Barbatis C, Zaharof A, Theodorou C. Is beta-human chorionic gonadotrophin production by transitional cell carcinoma of the bladder a marker of aggressive disease and resistance to radiotherapy? Br J Urol. 1993 Dec;72(6):907–9. doi: 10.1111/j.1464-410x.1993.tb16294.x. 7508330

[29] Dirnhofer S, Koessler P, Ensinger C, Feichtinger H, Madersbacher S, Berger P. Production of trophoblastic hormones by transitional cell carcinoma of the bladder: association to tumor stage and grade. Hum Pathol. 1998 Apr;29(4):377–82. doi: 10.1016/s0046-8177(98)90119-8. 9563788

[30] Przybycin CG, McKenney JK, Nguyen JK, Shah RB, Umar SA, Harik L, Shih IM, Cox RM. Urothelial Carcinomas With Trophoblastic Differentiation, Including Choriocarcinoma: Clinicopathologic Series of 16 Cases. Am J Surg Pathol. 2020 Oct;44(10):1322–1330. doi: 10.1097/PAS.0000000000001532. 32931680

[31] Iles RK, Oliver RT, Kitau M, Walker C, Chard T. In vitro secretion of human chorionic gonadotrophin by bladder tumour cells. Br J Cancer. 1987 Jun;55(6):623–6. doi: 10.1038/bjc.1987.126. 2441730 PMC2002027

[32] Fukutani K, Libby JM, Panko WB, Scardino PT. Human chorionic gonadotropin detected in urinary concentrates from patients with malignant tumors of the testis, prostate, bladder, ureter and kidney. J Urol. 1983 Jan;129(1):74–7. doi: 10.1016/s0022-5347(17)51926-6. 6827688

[33] Dexeus F, Logothetis C, Hossan E, Samuels ML. Carcinoembryonic antigen and beta-human chorionic gonadotropin as serum markers for advanced urothelial malignancies. J Urol. 1986 Aug;136(2):403–7. doi: 10.1016/s0022-5347(17)44882-8. 2426474

[34] Iles RK, Jenkins BJ, Oliver RT, Blandy JP, Chard T. Beta human chorionic gonadotrophin in serum and urine. A marker for metastatic urothelial cancer. Br J Urol. 1989 Sep;64(3):241–4. doi: 10.1111/j.1464-410x.1989.tb06006.x. 2478247

[35] Hotakainen K, Lintula S, Stenman J, Rintala E, Lindell O, Stenman UH. Detection of messenger RNA for the beta-subunit of chorionic gonadotropin in urinary cells from patients with transitional cell carcinoma of the bladder by reverse transcription-polymerase chain reaction. Int J Cancer. 1999 Jun 21;84(3):304–8. doi: 10.1002/(sici)1097-0215(19990621)84:3<304::aid-ijc18>3.0.co;2-b. 10371351

[36] Mariathasan S, Turley SJ, Nickles D, Castiglioni A, Yuen K, Wang Y, Kadel EE III, Koeppen H, Astarita JL, Cubas R, Jhunjhunwala S, Banchereau R, Yang Y, Guan Y, Chalouni C, Ziai J, Şenbabaoğlu Y, Santoro S, Sheinson D, Hung J, Giltnane JM, Pierce AA, Mesh K, Lianoglou S, Riegler J, Carano RAD, Eriksson P, Höglund M, Somarriba L, Halligan DL, van der Heijden MS, Loriot Y, Rosenberg JE, Fong L, Mellman I, Chen DS, Green M, Derleth C, Fine GD, Hegde PS, Bourgon R, Powles T. TGFβ attenuates tumour response to PD-L1 blockade by contributing to exclusion of T cells. Nature. 2018 Feb 22;554(7693):544–548. doi: 10.1038/nature25501. Epub 2018 Feb 14. 29443960 PMC6028240

[37] Zaretsky JM, Garcia-Diaz A, Shin DS, Escuin-Ordinas H, Hugo W, Hu-Lieskovan S, Torrejon DY, Abril-Rodriguez G, Sandoval S, Barthly L, Saco J, Homet Moreno B, Mezzadra R, Chmielowski B, Ruchalski K, Shintaku IP, Sanchez PJ, Puig-Saus C, Cherry G, Seja E, Kong X, Pang J, Berent-Maoz B, Comin-Anduix B, Graeber TG, Tumeh PC, Schumacher TN, Lo RS, Ribas A. Mutations Associated with Acquired Resistance to PD-1 Blockade in Melanoma. N Engl J Med. 2016 Sep 1;375(9):819–29. doi: 10.1056/NEJMoa1604958. Epub 2016 Jul 13. 27433843 PMC5007206

[38] Grasso CS, Tsoi J, Onyshchenko M, Abril-Rodriguez G, Ross-Macdonald P, Wind-Rotolo M, Champhekar A, Medina E, Torrejon DY, Shin DS, Tran P, Kim YJ, Puig-Saus C, Campbell K, Vega-Crespo A, Quist M, Martignier C, Luke JJ, Wolchok JD, Johnson DB, Chmielowski B, Hodi FS, Bhatia S, Sharfman W, Urba WJ, Slingluff CL Jr, Diab A, Haanen JBAG, Algarra SM Pardoll DM, Anagnostou V, Topalian SL, Velculescu VE, Speiser DE, Kalbasi A, Ribas A. Conserved Interferon-γ Signaling Drives Clinical Response to Immune Checkpoint Blockade Therapy in Melanoma. Cancer Cell. 2020 Oct 12;38(4):500–515.e3. doi: 10.1016/j.ccell.2020.08.005. Epub 2020 Sep 10. Erratum in: Cancer Cell. 2021 Jan 11;39(1):122. 32916126 PMC7872287

[39] Sjödahl G, Lauss M, Lövgren K, Chebil G, Gudjonsson S, Veerla S, Patschan O, Aine M, Fernö M, Ringnér M, Månsson W, Liedberg F, Lindgren D, Höglund M. A molecular taxonomy for urothelial carcinoma. Clin Cancer Res. 2012 Jun 15;18(12):3377–86. doi: 10.1158/1078-0432.CCR-12-0077-T. Epub 2012 May 2. 22553347

[40] Sjödahl G, Lövgren K, Lauss M, Patschan O, Gudjonsson S, Chebil G, Aine M, Eriksson P, Månsson W, Lindgren D, Fernö M, Liedberg F, Höglund M. Toward a molecular pathologic classification of urothelial carcinoma. Am J Pathol. 2013 Sep;183(3):681–91. doi: 10.1016/j.ajpath.2013.05.013. Epub 2013 Jul 1. 23827819

[41] Balar AV, Galsky MD, Rosenberg JE, Powles T, Petrylak DP, Bellmunt J, Loriot Y, Necchi A, Hoffman-Censits J, Perez-Gracia JL, Dawson NA, van der Heijden MS, Dreicer R, Srinivas S, Retz MM, Joseph RW, Drakaki A, Vaishampayan UN, Sridhar SS, Quinn DI, Durán I, Shaffer DR, Eigl BJ, Grivas PD, Yu EY, Li S, Kadel EE 3rd, Boyd Z, Bourgon R, Hegde PS, Mariathasan S, Thåström A, Abidoye OO, Fine GD, Bajorin DF; IMvigor210 Study Group. Atezolizumab as first-line treatment in cisplatin-ineligible patients with locally advanced and metastatic urothelial carcinoma: a single-arm, multicentre, phase 2 trial. Lancet. 2017 Jan 7;389(10064):67–76. doi: 10.1016/S0140-6736(16)32455-2. Epub 2016 Dec 8. Erratum in: Lancet. 2017 Aug 26;390(10097):848. doi: 10.1016/S0140–6736(17)32213–4. 27939400 PMC5568632

[42] Samstein RM, Lee CH, Shoushtari AN, Hellmann MD, Shen R, Janjigian YY, Barron DA, Zehir A, Jordan EJ, Omuro A, Kaley TJ, Kendall SM, Motzer RJ, Hakimi AA, Voss MH, Russo P, Rosenberg J, Iyer G, Bochner BH, Bajorin DF, Al-Ahmadie HA, Chaft JE, Rudin CM, Riely GJ, Baxi S, Ho AL, Wong RJ, Pfister DG, Wolchok JD, Barker CA, Gutin PH, Brennan CW, Tabar V, Mellinghoff IK, DeAngelis LM, Ariyan CE, Lee N, Tap WD, Gounder MM, D’Angelo SP, Saltz L, Stadler ZK, Scher HI, Baselga J, Razavi P, Klebanoff CA, Yaeger R, Segal NH, Ku GY, DeMatteo RP, Ladanyi M, Rizvi NA, Berger MF, Riaz N, Solit DB, Chan TA, Morris LGT. Tumor mutational load predicts survival after immunotherapy across multiple cancer types. Nat Genet. 2019 Feb;51(2):202–206. doi: 10.1038/s41588-018-0312-8. Epub 2019 Jan 14. 30643254 PMC6365097

[43] Ricciuti B, Wang X, Alessi JV, Rizvi H, Mahadevan NR, Li YY, Polio A, Lindsay J, Umeton R, Sinha R, Vokes NI, Recondo G, Lamberti G, Lawrence M, Vaz VR, Leonardi GC, Plodkowski AJ, Gupta H, Cherniack AD, Tolstorukov MY, Sharma B, Felt KD, Gainor JF, Ravi A, Getz G, Schalper KA, Henick B, Forde P, Anagnostou V, Jänne PA, Van Allen EM, Nishino M, Sholl LM, Christiani DC, Lin X, Rodig SJ, Hellmann MD, Awad MM. Association of High Tumor Mutation Burden in Non-Small Cell Lung Cancers With Increased Immune Infiltration and Improved Clinical Outcomes of PD-L1 Blockade Across PD-L1 Expression Levels. JAMA Oncol. 2022 Aug 1;8(8):1160–1168. doi: 10.1001/jamaoncol.2022.1981. Erratum in: JAMA Oncol. 2022 Nov 1;8(11):1702. doi: 10.1001/jamaoncol.2022.5957. 35708671 PMC9204620

[44] Hellmann MD, Paz-Ares L, Bernabe Caro R, Zurawski B, Kim SW, Carcereny Costa E, Park K, Alexandru A, Lupinacci L, de la Mora Jimenez E, Sakai H, Albert I, Vergnenegre A, Peters S, Syrigos K, Barlesi F, Reck M, Borghaei H, Brahmer JR, O’Byrne KJ, Geese WJ, Bhagavatheeswaran P, Rabindran SK, Kasinathan RS, Nathan FE, Ramalingam SS. Nivolumab plus Ipilimumab in Advanced Non-Small-Cell Lung Cancer. N Engl J Med. 2019 Nov 21;381(21):2020–2031. doi: 10.1056/NEJMoa1910231. Epub 2019 Sep 28. 31562796

[45] Hellmann MD, Ciuleanu TE, Pluzanski A, Lee JS, Otterson GA, Audigier-Valette C, Minenza E, Linardou H, Burgers S, Salman P, Borghaei H, Ramalingam SS, Brahmer J, Reck M, O’Byrne KJ, Geese WJ, Green G, Chang H, Szustakowski J, Bhagavatheeswaran P, Healey D, Fu Y, Nathan F, Paz-Ares L. Nivolumab plus Ipilimumab in Lung Cancer with a High Tumor Mutational Burden. N Engl J Med. 2018 May 31;378(22):2093–2104. doi: 10.1056/NEJMoa1801946. Epub 2018 Apr 16. 29658845 PMC7193684

[46] Ye Y, Jing Y, Li L, Mills GB, Diao L, Liu H, Han L. Sex-associated molecular differences for cancer immunotherapy. Nat Commun. 2020 Apr 14;11(1):1779. doi: 10.1038/s41467-020-15679-x. 32286310 PMC7156379

[47] Jang SR, Nikita N, Banks J, Keith SW, Johnson JM, Wilson M, Lu-Yao G. Association Between Sex and Immune Checkpoint Inhibitor Outcomes for Patients With Melanoma. JAMA Netw Open. 2021 Dec 1;4(12):e2136823. doi: 10.1001/jamanetworkopen.2021.36823. 34854905 PMC8640892

[48] Ishwaran Hemant, Kogalur Udaya B., Blackstone Eugene H., Lauer Michael S.. “Random survival forests.” The Annals of Applied Statistics, 2(3) 841–860 September 2008. 10.1214/08-AOAS169

[49] Ishwaran H, Malley JD. Synthetic learning machines. BioData Min. 2014 Dec 18;7(1):28. doi: 10.1186/s13040-014-0028-y. PMC4279689 25614764

[50] Pineda JMB, Bradley RK. DUX4 is a common driver of immune evasion and immunotherapy failure in metastatic cancers. Elife. 2024 Jun 3;12:RP89017. doi: 10.7554/eLife.89017. PMC11147511 38829686

[51] Pickett KL, Suresh K, Campbell KR, Davis S, Juarez-Colunga E. Random survival forests for dynamic predictions of a time-to-event outcome using a longitudinal biomarker. BMC Med Res Methodol. 2021 Oct 17;21(1):216. doi: 10.1186/s12874-021-01375-x. 34657597 PMC8520610

[52] Zhao B, Nguyen VK, Xu M, Colacino JA, Jolliet O. Random survival forest for predicting the combined effects of multiple physiological risk factors on all-cause mortality. Sci Rep. 2024 Jul 6;14(1):15566. doi: 10.1038/s41598-024-66261-0. 38971926 PMC11227534

[53] Sarica A, Aracri F, Bianco MG, Arcuri F, Quattrone A, Quattrone A; Alzheimer’s Disease Neuroimaging Initiative. Explainability of random survival forests in predicting conversion risk from mild cognitive impairment to Alzheimer’s disease. Brain Inform. 2023 Nov 18;10(1):31. doi: 10.1186/s40708-023-00211-w. 37979033 PMC10657350

[54] Bohannan ZS, Coffman F, Mitrofanova A. Random survival forest model identifies novel biomarkers of event-free survival in high-risk pediatric acute lymphoblastic leukemia. Comput Struct Biotechnol J. 2022 Jan 6;20:583–597. doi: 10.1016/j.csbj.2022.01.003. 35116134 PMC8777142

[55] Jung SY, Papp JC, Sobel EM, Yu H, Zhang ZF. Breast Cancer Risk and Insulin Resistance: Post Genome-Wide Gene-Environment Interaction Study Using a Random Survival Forest. Cancer Res. 2019 May 15;79(10):2784–2794. doi: 10.1158/0008-5472.CAN-18-3688. Epub 2019 Apr 1. 30936085 PMC6522308

[56] Maksymiuk AW, Tappia PS, Bux RA, Moyer D, Huang G, Joubert P, Miller DW, Ramjiawan B, Sitar DS. Use of amantadine in the evaluation of response to chemotherapy in lung cancer: a pilot study. Future Sci OA. 2021 Jan 27;7(4):FSO679. doi: 10.2144/fsoa-2020-0176. 33815824 PMC8015664

[57] Shapley L. S. (1953). A Value for n-Person Games. Contributions to the Theory of Games, 2, 307–317. 10.1515/9781400881970-018

[58] Štrumbelj E., Kononenko I. 2014. Explaining prediction models and individual predictions with feature contributions. Knowledge and Information Systems 41:647–665. 10.1007/s10115-013-0679-x

[59] Flicek P, Ahmed I, Amode MR, Barrell D, Beal K, Brent S, Carvalho-Silva D, Clapham P, Coates G, Fairley S, Fitzgerald S, Gil L, García-Girón C, Gordon L, Hourlier T, Hunt S, Juettemann T, Kähäri AK, Keenan S, Komorowska M, Kulesha E, Longden I, Maurel T, McLaren WM, Muffato M, Nag R, Overduin B, Pignatelli M, Pritchard B, Pritchard E, Riat HS, Ritchie GR, Ruffier M, Schuster M, Sheppard D, Sobral D, Taylor K, Thormann A, Trevanion S, White S, Wilder SP, Aken BL, Birney E, Cunningham F, Dunham I, Harrow J, Herrero J, Hubbard TJ, Johnson N, Kinsella R, Parker A, Spudich G, Yates A, Zadissa A, Searle SM. Ensembl 2013. Nucleic Acids Res. 2013 Jan;41(Database issue):D48–55. doi: 10.1093/nar/gks1236. Epub 2012 Nov 30. 23203987 PMC3531136

[60] Meyer LR, Zweig AS, Hinrichs AS, Karolchik D, Kuhn RM, Wong M, Sloan CA, Rosenbloom KR, Roe G, Rhead B, Raney BJ, Pohl A, Malladi VS, Li CH, Lee BT, Learned K, Kirkup V, Hsu F, Heitner S, Harte RA, Haeussler M, Guruvadoo L, Goldman M, Giardine BM, Fujita PA, Dreszer TR, Diekhans M, Cline MS, Clawson H, Barber GP, Haussler D, Kent WJ. The UCSC Genome Browser database: extensions and updates 2013. Nucleic Acids Res. 2013 Jan;41(Database issue):D64–9. doi: 10.1093/nar/gks1048. Epub 2012 Nov 15. 23155063 PMC3531082

[61] Katz Y, Wang ET, Airoldi EM, Burge CB. Analysis and design of RNA sequencing experiments for identifying isoform regulation. Nat Methods. 2010 Dec;7(12):1009–15. doi: 10.1038/nmeth.1528. Epub 2010 Nov 7. 21057496 PMC3037023

[62] Li B, Dewey CN. RSEM: accurate transcript quantification from RNA-Seq data with or without a reference genome. BMC Bioinformatics. 2011 Aug 4;12:323. doi: 10.1186/1471-2105-12-323. 21816040 PMC3163565

[63] Langmead B, Trapnell C, Pop M, Salzberg SL. Ultrafast and memory-efficient alignment of short DNA sequences to the human genome. Genome Biol. 2009;10(3):R25. doi: 10.1186/gb-2009-10-3-r25. Epub 2009 Mar 4. 19261174 PMC2690996

[64] Trapnell C, Pachter L, Salzberg SL. TopHat: discovering splice junctions with RNA-Seq. Bioinformatics. 2009 May 1;25(9):1105–11. doi: 10.1093/bioinformatics/btp120. Epub 2009 Mar 16. 19289445 PMC2672628

[65] Robinson MD, Oshlack A. A scaling normalization method for differential expression analysis of RNA-seq data. Genome Biol. 2010;11(3):R25. doi: 10.1186/gb-2010-11-3-r25. Epub 2010 Mar 2. 20196867 PMC2864565

[66] Therneau T (2024). A Package for Survival Analysis in R. R package version 3.7–0, https://CRAN.R-project.org/package=survival.

[67] Greenwell B (2024). fastshap: Fast Approximate Shapley Values. R package version 0.1.1, https://bgreenwell.github.io/fastshap/, https://github.com/bgreenwell/fastshap.

[68] Wickham H, François R, Henry L, Müller K, Vaughan D (2023). dplyr: A Grammar of Data Manipulation. R package version 1.1.4, https://github.com/tidyverse/dplyr, https://dplyr.tidyverse.org.

[69] Wickham H (2016). ggplot2: Elegant Graphics for Data Analysis. Springer-Verlag New York. ISBN 978–3-319–24277-4, https://ggplot2.tidyverse.org.

[70] Bender A, Scheipl F (2018). “pammtools: Piece-wise exponential Additive Mixed Modeling tools.” arXiv:1806.01042 [stat]. https://arxiv.org/abs/1806.01042.

[71] Sievert C (2020). Interactive Web-Based Data Visualization with R, plotly, and shiny. Chapman and Hall/CRC. ISBN 9781138331457, https://plotly-r.com.

[72] Blanche P, Dartigues J, Jacqmin-Gadda H (2013). “Estimating and Comparing time-dependent areas under receiver operating characteristic curves for censored event times with competing risks.” Statistics in Medicine, 32(30), 5381–5397. http://onlinelibrary.wiley.com/doi/10.1002/sim.5958/full. 24027076 10.1002/sim.5958

[73] Moulton HM, Yoshihara PH, Mason DH, Iversen PL, Triozzi PL. Active specific immunotherapy with a beta-human chorionic gonadotropin peptide vaccine in patients with metastatic colorectal cancer: antibody response is associated with improved survival. Clin Cancer Res. 2002 Jul;8(7):2044–51. 12114402

