# Supplementary material for: Chorionic Gonadotropin Beta 7 is a marker of immune evasion in cancer
Source: bioRxiv. 2025 Jun 2:2025.05.28.656535. Preprint. [Version 1] doi: 10.1101/2025.05.28.656535 (PMC12157434; doi:10.1101/2025.05.28.656535)
Supplement: 1 [file NIHPP2025.05.28.656535V1-supplement-1.pdf]

bioRxiv preprint doi: https://doi.org/10.1101/2025.05.28.656535; this version posted June 2, 2025. The copyright holder for this preprint (which was not certified by peer review) is the author/funder, who has granted bioRxiv a license to display the preprint in perpetuity. It is made available under aCC-BY 4.0 International license.

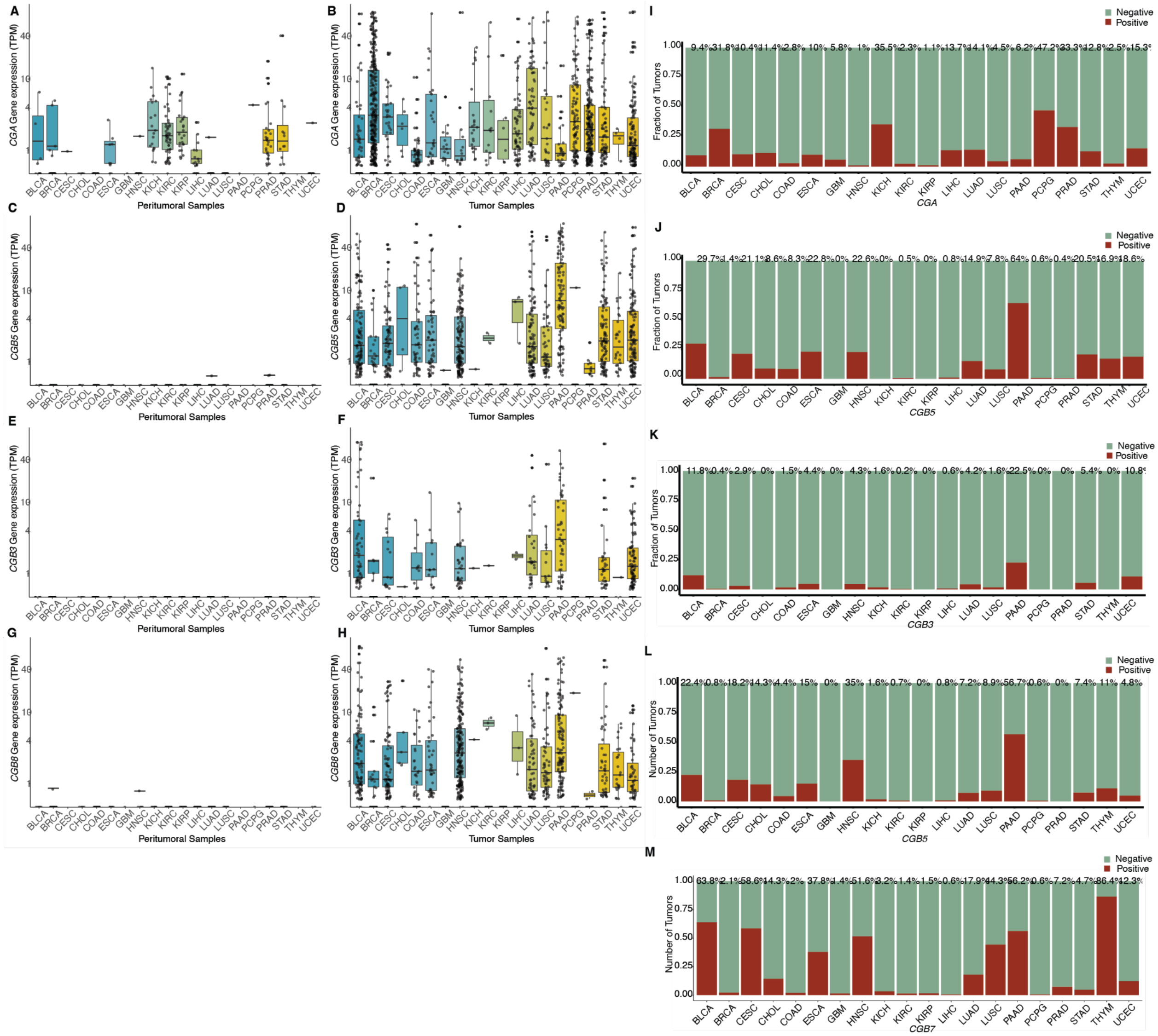

Supplemental Figure 1: CGB is expressed in multiple cancer types.

CGA mRNA expression in healthy peritumoral tissue samples (**A**) and matched tumor tissue samples (**B**) across 20 cancer types from the TCGA. Cancer type reflects the site of the primary tumor.

CGB3 mRNA expression in healthy peritumoral tissue samples (**C**) and matched tumor tissue samples (**D**) across 20 cancer types from the TCGA. Cancer type reflects the site of the primary tumor.

CGB5 mRNA expression in healthy peritumoral tissue samples (**E**) and matched tumor tissue samples (**F**) across 20 cancer types from the TCGA. Cancer type reflects the site of the primary tumor.

CGB8 mRNA expression in healthy peritumoral tissue samples (**G**) and matched tumor tissue samples (**H**) across 20 cancer types from the TCGA. Cancer type reflects the site of the primary tumor.

bioRxiv preprint doi: https://doi.org/10.1101/2025.05.28.656535; this version posted June 2, 2025. The copyright holder for this preprint (which was not certified by peer review) is the author/funder, who has granted bioRxiv a license to display the preprint in perpetuity. It is made available under aCC-BY 4.0 International license.

(**I, J, K, L, M**) Fraction of tumors across 20 cancer types expressing *CGA*, *CGB3*, *CGB5*, *CGB8*, or *CGB7*, gene expression with TPM >1, respectively. Percent positive is annotated above each cancer type.

TPM = transcripts per million.

bioRxiv preprint doi: https://doi.org/10.1101/2025.05.28.656535; this version posted June 2, 2025. The copyright holder for this preprint (which was not certified by peer review) is the author/funder, who has granted bioRxiv a license to display the preprint in perpetuity. It is made available under aCC-BY 4.0 International license.

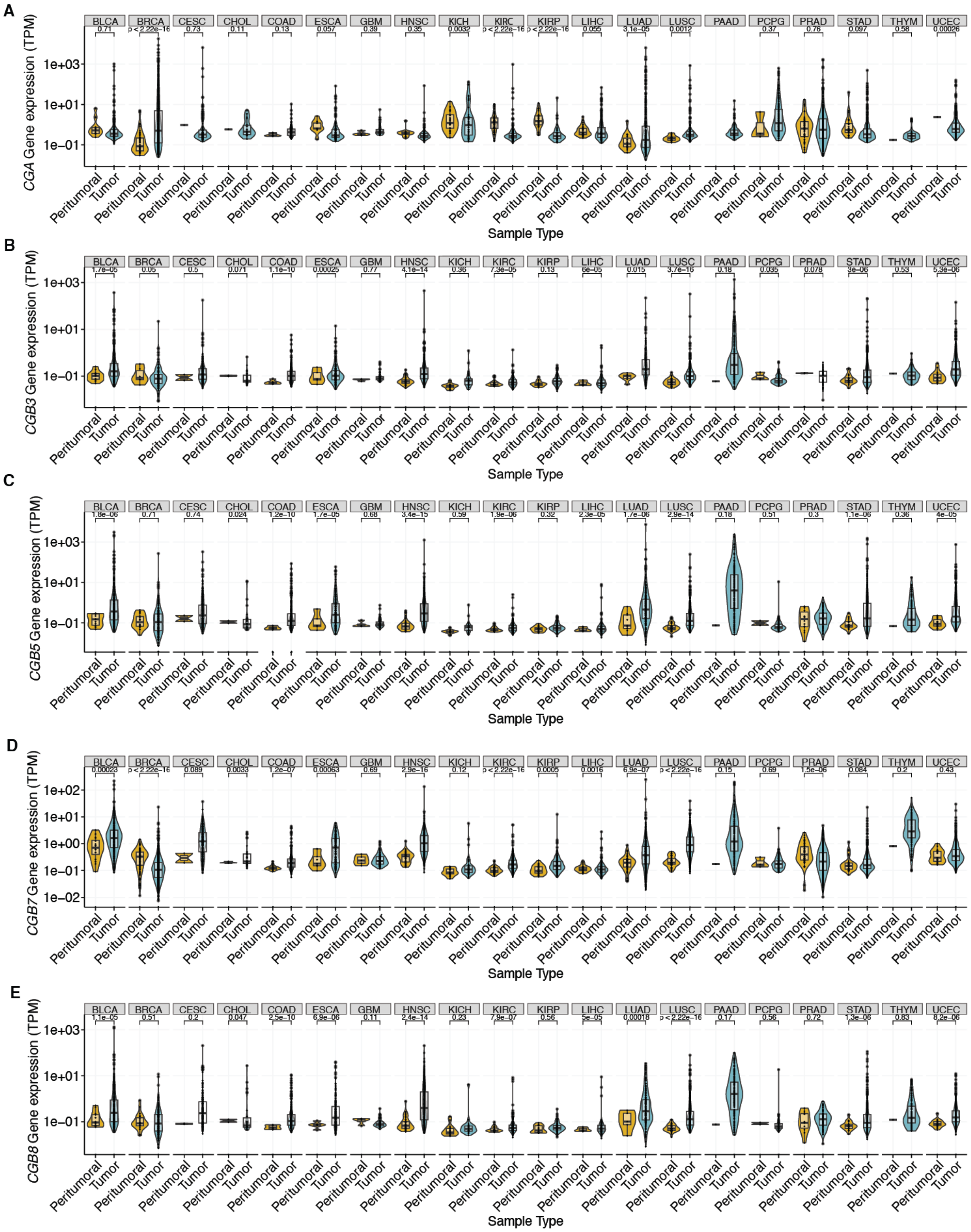

Supplemental Figure 2: CGB expression is upregulated across cancer types.

bioRxiv preprint doi: https://doi.org/10.1101/2025.05.28.656535; this version posted June 2, 2025. The copyright holder for this preprint (which was not certified by peer review) is the author/funder, who has granted bioRxiv a license to display the preprint in perpetuity. It is made available under aCC-BY 4.0 International license.

Violin plots of *CGA* (**A**), *CGB3* (**B**), *CGB5* (**C**), *CGB7* (**D**), or *CGB8* (**E**) expression in tumors and matched healthy peritumoral tissue samples across cancer type datasets from The Cancer Genome Atlas. Expression is in transcripts per million (TPM). P-values determined by wilcox signed rank test.

bioRxiv preprint doi: https://doi.org/10.1101/2025.05.28.656535; this version posted June 2, 2025. The copyright holder for this preprint (which was not certified by peer review) is the author/funder, who has granted bioRxiv a license to display the preprint in perpetuity. It is made available under aCC-BY 4.0 International license.

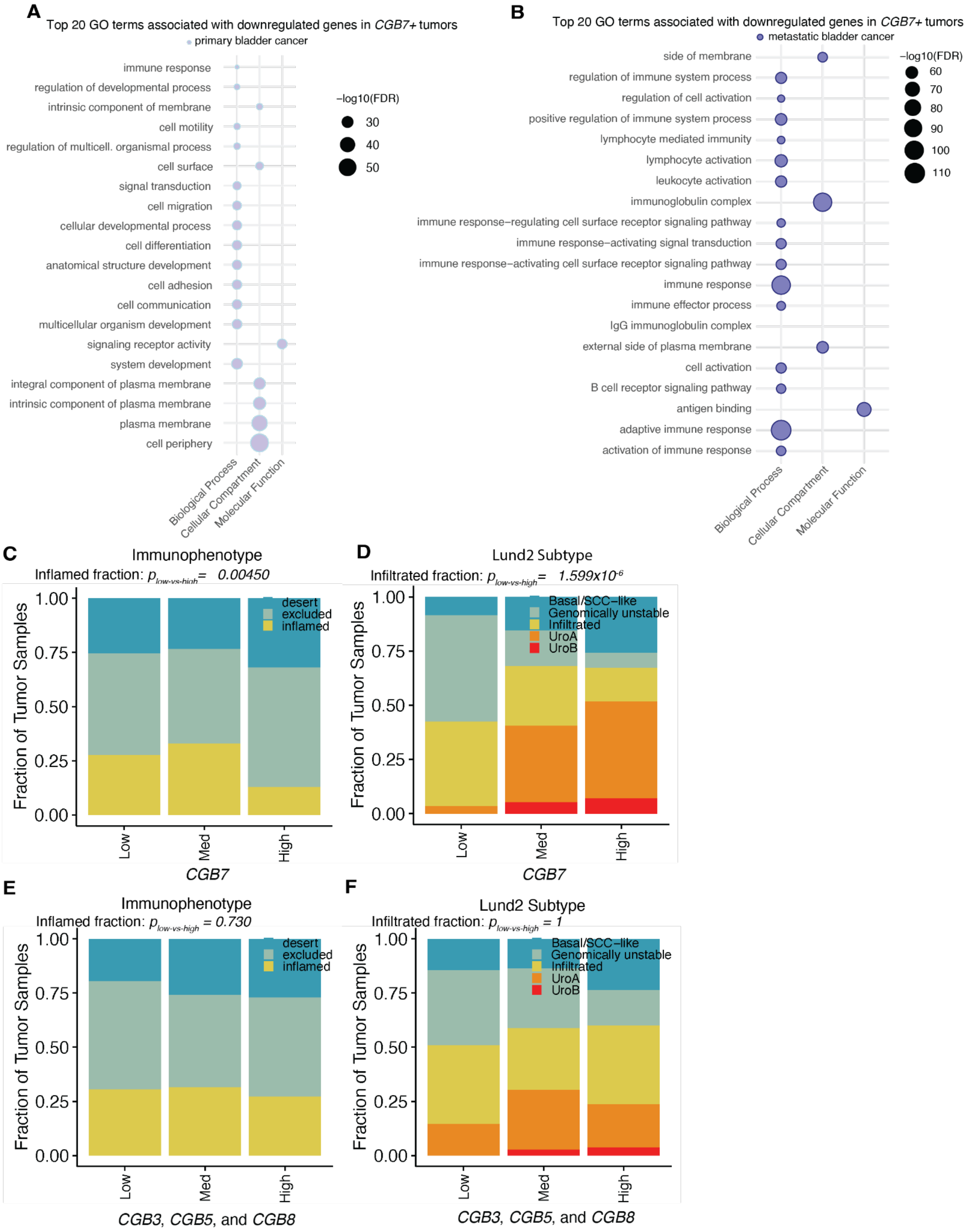

Supplemental Figure 3: CGB expression is associated with altered immune infiltrate.
(**A**) The top 20 Gene Ontology terms associated with *CGB7* expression in primary urothelial cancers in the TCGA BLCA dataset.
(**B**) The top 20 Gene Ontology terms associated with *CGB7* expression in metastatic urothelial cancers in the IMVigor 210 clinical trial dataset (data re-analyzed from Mariathasan et al., 2018).

bioRxiv preprint doi: https://doi.org/10.1101/2025.05.28.656535; this version posted June 2, 2025. The copyright holder for this preprint (which was not certified by peer review) is the author/funder, who has granted bioRxiv a license to display the preprint in perpetuity. It is made available under aCC-BY 4.0 International license.

(**C**) Immunophenotype data: immune desert, immune excluded, or inflamed. Tumors with expression of *CGB3*, *CGB5*, or *CGB8* > 1 TPM were removed, and remaining samples stratified by *CGB7* expression. P-values determined by proportions test with continuity correction. Chi-squared = 8.072, df = 1, p-value = 0.0045.
(**D**) Tumor subtype data: basal/SCC-like, genomically unstable, immune infiltrated, urothelial type A, or urothelial type B. Tumors with expression of *CGB3*, *CGB5*, or *CGB8* > 1 TPM were removed, and remaining samples stratified by *CGB7* expression. P-values determined by proportions test with continuity correction. Chi-squared = 23.025, df = 1, p-value = 1.60×10^{-6}.
(**E**) Immunophenotype data: immune desert, immune excluded, or inflamed. Tumors with expression of *CGB3*, *CGB5*, or *CGB8* > 1 TPM were removed, and remaining samples stratified by *CGB7* expression. P-values determined by proportions test with continuity correction. Chi-squared = 0.119, df = 1, p-value = 0.730.
(**F**) Tumor subtype data: basal/SCC-like, genomically unstable, immune infiltrated, urothelial type A, or urothelial type B. Tumors with expression of *CGB7* > 1 TPM were removed, and remaining samples stratified by summed *CGB3*, *CGB5*, and *CGB8* expression. P-values determined by proportions test with continuity correction. Chi-squared = 0, df = 1, p-value = 1.

Med = medium.

bioRxiv preprint doi: https://doi.org/10.1101/2025.05.28.656535; this version posted June 2, 2025. The copyright holder for this preprint (which was not certified by peer review) is the author/funder, who has granted bioRxiv a license to display the preprint in perpetuity. It is made available under aCC-BY 4.0 International license.

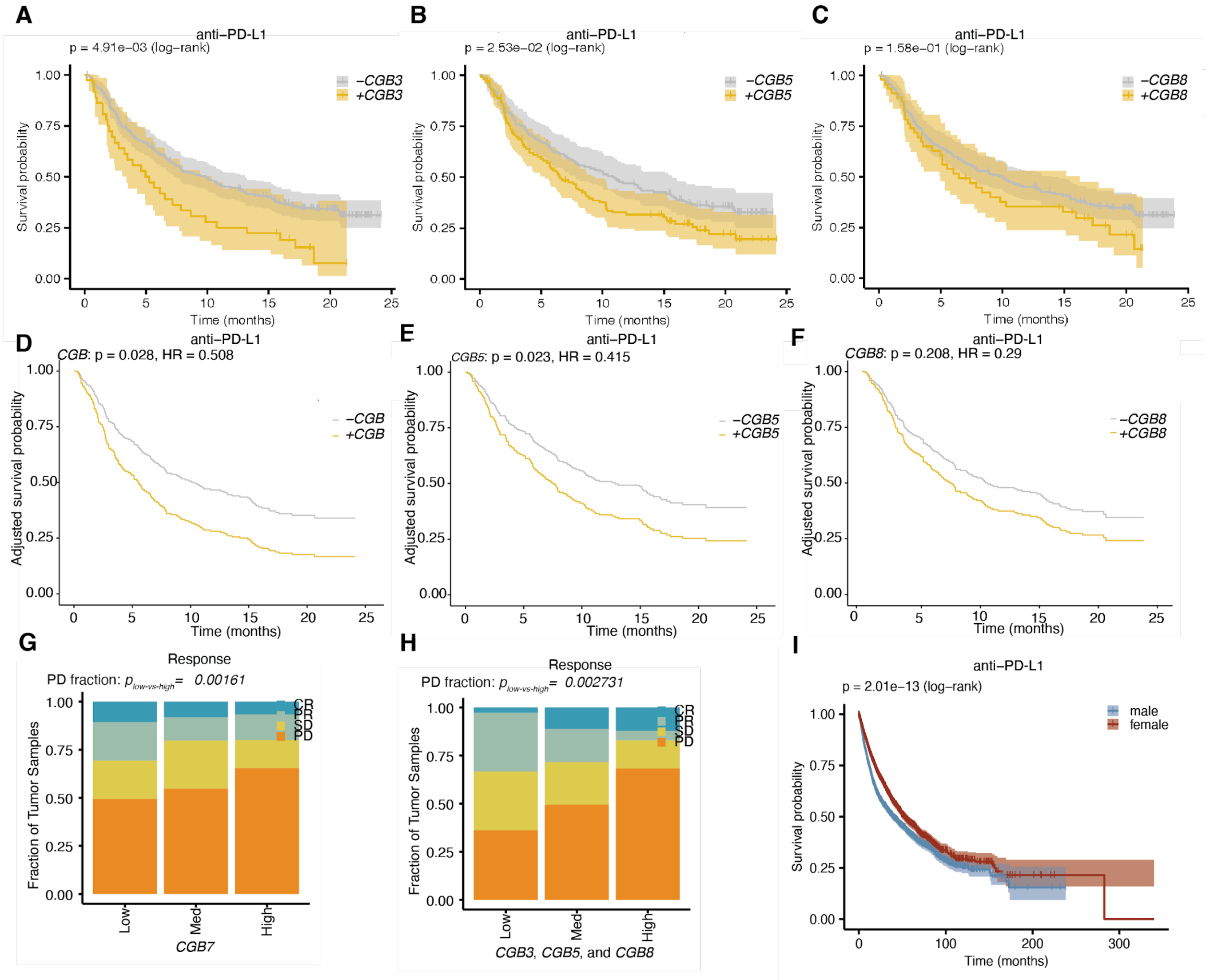

Figure S4: CGB expression is associated with decreased response to ICI therapy.

(**A**) Kaplan Meier overall survival curves comparing patients with *CGB3+* (gold) and *CGB3-* (gray) advanced urothelial cancer tumors (data re-analyzed from Mariathasan et al., 2018). All patients received Atezolizumab (anti-PD-L1). P-value determined by log rank test.

(**B**) As in (**A**), but comparing patients with *CGB5* positive (gold) and negative (gray) tumors.

(**C**) As in (**A**), but comparing patients with *CGB8* positive (gold) and negative (gray) tumors.

(**D**) Kaplan Meier overall survival curves as shown in (**A**) adjusted for confounding effects of sex and tumor mutational burden covariates by cox proportional hazards modeling. Hazard ratio (HR) and p-value obtained from fitting a cox proportional hazards model.

(**E**) Kaplan Meier overall survival curves as shown in (**B**) adjusted as described in (**D**).

(**F**) Kaplan Meier overall survival curves as shown in (**C**) adjusted as described in (**D**).

(**G**) Response determined by RECIST (Mariathasan 2018). Tumors with expression of *CGB3*, *CGB5*, or *CGB8* > 1 TPM were removed, and remaining samples stratified by *CGB7* expression. P-values determined by proportions test with continuity correction. Chi-squared = 9.954, df = 1, p-value = 0.00161.

bioRxiv preprint doi: https://doi.org/10.1101/2025.05.28.656535; this version posted June 2, 2025. The copyright holder for this preprint (which was not certified by peer review) is the author/funder, who has granted bioRxiv a license to display the preprint in perpetuity. It is made available under aCC-BY 4.0 International license.

(**H**) Response determined by RECIST (Mariathasan et al., 2018). Tumors with expression of *CGB7* > 1 TPM were removed, and remaining samples stratified by summed *CGB3*, *CGB5*, and *CGB8* expression. P-values determined by proportions test with continuity correction. Chi-squared = 8.979, df = 1, p-value = 0.00273.
(**I**) Kaplan Meier overall survival curves comparing male (blue) and female (red) patients with advanced urothelial carcinoma tumors. All patients received Atezolizumab (anti-PD-L1). P-value determined by log rank test.

Response Evaluation Criteria in Solid Tumors (RECIST) scoring: CR = complete response, PR = partial response, SD = stable disease, PD = progressive disease. Med = medium.

bioRxiv preprint doi: https://doi.org/10.1101/2025.05.28.656535; this version posted June 2, 2025. The copyright holder for this preprint (which was not certified by peer review) is the author/funder, who has granted bioRxiv a license to display the preprint in perpetuity. It is made available under aCC-BY 4.0 International license.

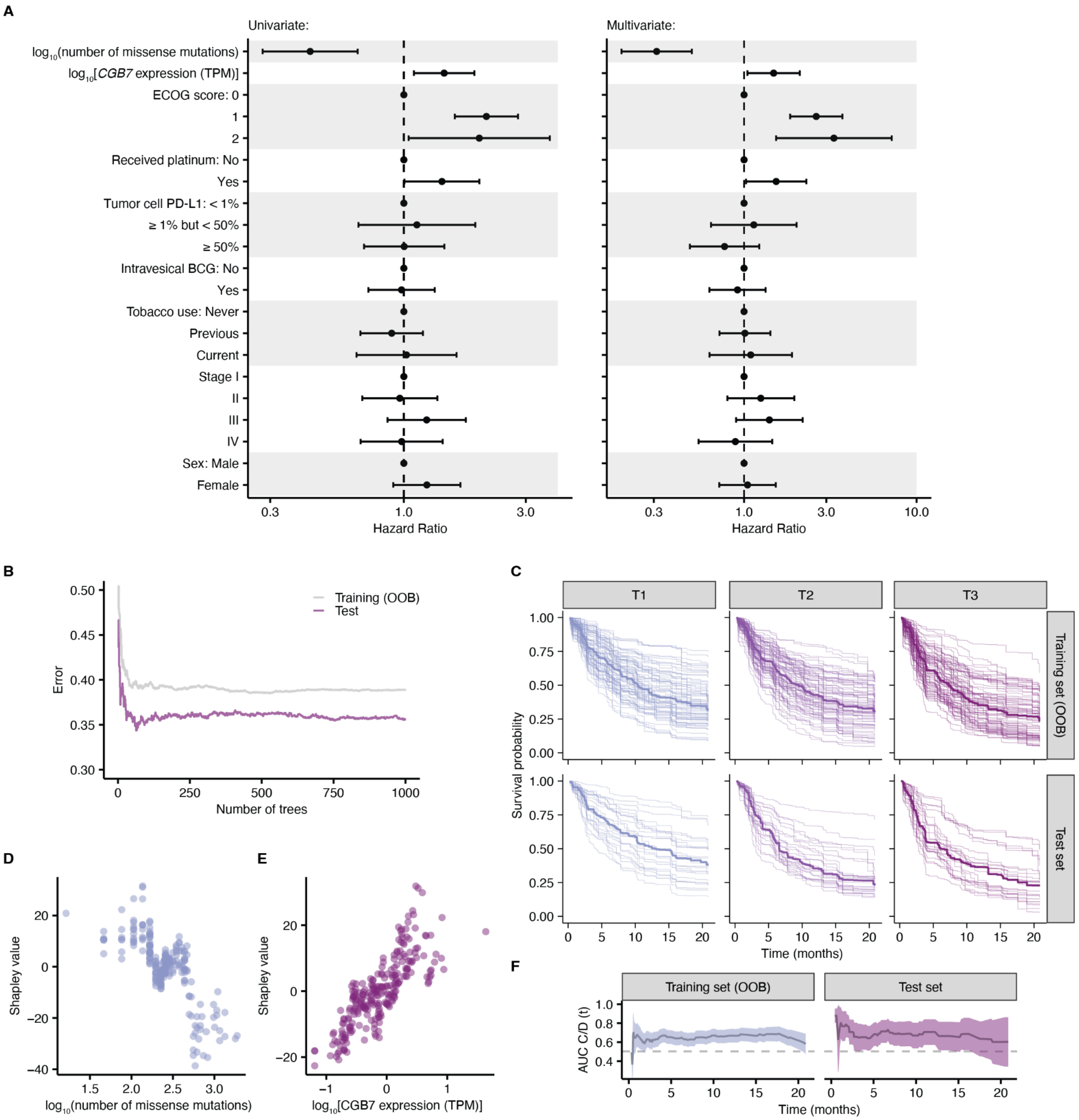

Figure S5: Statistical and machine learning models exhibit the negative effects of *CGB7* expression on overall survival.

(**A**) Hazard ratios estimated by Cox Proportional Hazards Regression in the univariate (left) and multivariate (right) contexts. Error bars denote the 95% confidence interval of the hazard ratio. (**B**) The training out-of-bag error (OOB error, solid gray line) and the test error (solid purple line) as a function of the number of trees in the Random Survival Forest (RSF) model. Error is defined as 1 − Harrell's concordance index.

bioRxiv preprint doi: https://doi.org/10.1101/2025.05.28.656535; this version posted June 2, 2025. The copyright holder for this preprint (which was not certified by peer review) is the author/funder, who has granted bioRxiv a license to display the preprint in perpetuity. It is made available under aCC-BY 4.0 International license.

(**C**) RSF predicted overall survival for individual patients (thin lines) stratified into terciles, by *CGB7* expression. OOB survival predictions are shown for the patients in the training set. The median survival function across the cohort is shown (thick line).
(**D**) Shapley dependence plot correlating tumor mutational burden (TMB, number of missense mutations) and RSF mortality. Each patient is represented by a single point.
(**E**) As in (**D**), but showing *CGB7* expression.
(**F**) Time-dependent receiver operating characteristic (ROC) analyses. The cumulative/dynamic area under the ROC curve ($AUC^{C/D}$) (solid line) and associated 95% confidence interval (transparent ribbon) are calculated for the training and test sets. The training set $AUC^{C/D}$ was calculated using the RSF OOB mortality predictions.
